# Metabolic Reprogramming in Recurrent Spontaneous Abortion: Key Biomarkers Identification and Diagnostic Model Development

**DOI:** 10.1049/syb2.70078

**Published:** 2026-06-17

**Authors:** Fan Wu, Chuanmei Qin, Xiaowei Wei, Qian Li, Yi Yuan, Weihong Zeng, Yi Lin

**Affiliations:** ^1^ International Peace Maternity and Child Health Hospital Shanghai Jiao Tong University School of Medicine Shanghai China; ^2^ Shanghai Key Laboratory of Embryo Original Diseases Shanghai China; ^3^ Reproductive Medicine Center Shanghai Sixth People's Hospital Shanghai Jiao Tong University School of Medicine Shanghai China

**Keywords:** diagnostic model, immune microenvironment, metabolic reprogramming, recurrent spontaneous abortion, single‐cell analysis

## Abstract

Recurrent spontaneous abortion (RSA) involves complex and often unexplained pathogenesis. This study investigated metabolic reprogramming in RSA to identify diagnostic biomarkers. We integrated GEO datasets (GSE26787, GSE165004), identified metabolic reprogramming‐related differentially expressed genes, and intersected them with WGCNA modules. Module genes were refined using logistic regression, SVM‐RFE, and LASSO to construct a diagnostic model validated by ROC, nomogram, calibration, and decision curve analysis. The role of *SREBF2* was explored by PCR, western blot, CCK‐8, and transwell assays in vitro. Immune infiltration (ssGSEA, CIBERSORT) and single‐cell RNA sequencing (GSE214607) explored immune‐metabolic crosstalk. Results demonstrate critical involvement of metabolic reprogramming in RSA. Four model genes (*SREBF2*, *PPARG*, *SQLE*, *UCP2*) were upregulated in RSA and enriched in lipid/cholesterol pathways. Experimental validation confirmed *SREBF2* upregulation in RSA decidua. Its overexpression in endometrial stromal cells impaired cell proliferation, decidualization, and trophoblast migration. Immune infiltration revealed correlations between model genes and specific immune subsets. Single‐cell analysis identified altered immune cell proportions and cell‐type‐specific expression patterns, predominantly in macrophage subsets. This study indicates that lipid‐centric metabolic remodelling may concurrently affect decidual function, trophoblast behaviour, and immune tolerance in RSA, offering diagnostic and therapeutic insights.

## Graphical abstract

This study aims to identify differentially expressed genes associated with metabolic reprogramming in RSA. Using both bulk and single‐cell RNA sequencing datasets from the Gene Expression Omnibus (GEO), we constructed a machine learning‐based diagnostic model and investigated cell‐type‐specific immune‐metabolic crosstalk. Furthermore, experimental validation was conducted to elucidate the functional role of the core gene SREBF2 in RSA pathogenesis.

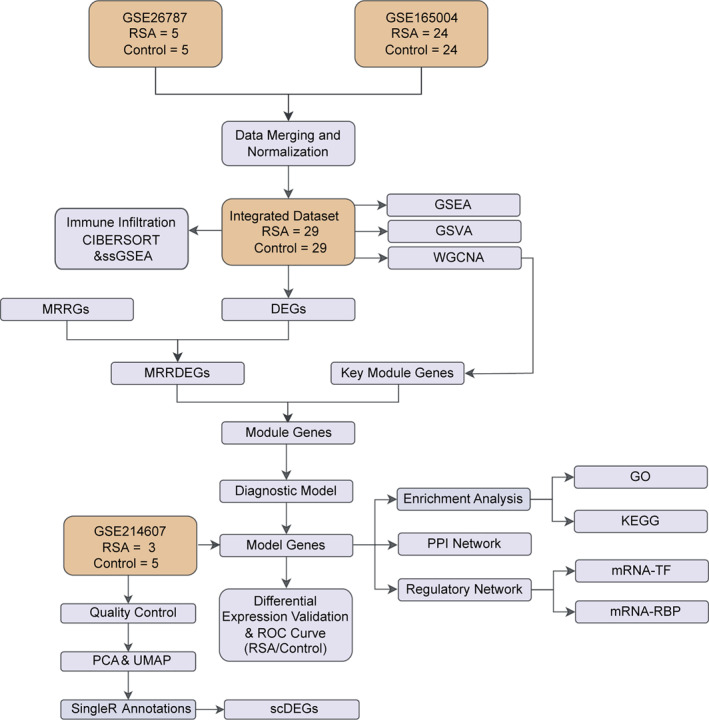

## 1 Introduction

Recurrent spontaneous abortion (RSA) is defined as having two or more failed clinical pregnancies. The causes of RSA are diverse, including genetic, endocrine, and immune factors, complicating both diagnosis and treatment [1]. Notably, up to 50% of RSA cases lack a clearly defined cause, which presents significant emotional and physical challenges for those affected [2, 3]. Current treatments, such as hormonal therapies and immunological interventions, often show limited success. This highlights the urgent need for new strategies to better understand and manage RSA.

Metabolic reprogramming has emerged as a critical area of research in understanding various diseases. Metabolic reprogramming refers to the adaptive changes in cellular metabolism that occur in response to different physiological and pathological conditions, influencing cell growth, proliferation and survival [4]. Studies have shown that metabolic alterations play a critical role in embryo implantation and the maintenance of pregnancy, particularly in cases of RSA [5]. Dysregulation in key metabolic pathways can disrupt the balance between cellular apoptosis and survival, leading to impaired placental development. Proper metabolic reprogramming helps prevent early placental cell apoptosis, thereby supporting pregnancy continuation [6]. Moreover, such reprogramming influences immune cell metabolism, for example, modulating monocyte function to enhance tolerance toward trophoblasts and reduce maternal immune rejection [7]. It also regulates the production of inflammatory mediators, mitigating detrimental pro‐inflammatory responses that may compromise pregnancy [8]. Additionally, metabolic reprogramming contributes to maintaining the redox equilibrium within the placental environment, shaping trophoblast responses to hypoxic conditions during early gestation [9]. A deeper understanding of RSA‐specific metabolic changes may offer valuable insights for developing targeted therapeutic strategies to improve pregnancy outcomes in affected women.

In this study, we identified differentially expressed genes associated with metabolic reprogramming in RSA. Using both bulk and single‐cell RNA sequencing datasets from the Gene Expression Omnibus (GEO), we constructed a machine learning‐based diagnostic model and investigated cell‐type‐specific immune‐metabolic crosstalk. The pathways enriched by the model genes converged on lipid metabolism and cholesterol homoeostasis. Furthermore, experimental validation was conducted to elucidate the functional role of the core gene *SREBF2* in RSA pathogenesis, involving decidual dysfunction and trophoblast abnormalities. This research aims to advance our understanding of the molecular mechanisms underlying RSA and to develop a reliable diagnostic tool that may ultimately improve clinical management for affected patients. We propose that a coordinated, lipid metabolism‐centred metabolic remodelling exists at the maternal‐foetal interface in RSA and may participate in regulating decidual activity, trophoblast behaviour, and immune tolerance.

## 2 Materials and Methods

### 2.1 Data Download

Through the R package GEOquery [10] (version 2.70.0), we downloaded datasets including GSE214607 [11], GSE26787 [12] and GSE165004 [13] from GEO database [14] (https://www.ncbi.nlm.nih.gov/geo/). All samples in these datasets are derived from *Homo sapiens* as detailed in Table 1.

**TABLE 1:** GEO datasets information.

	GSE214607	GSE26787	GSE165004
Platform	GPL24676	GPL570	GPL16699
Species	*Homo sapiens*	*H. sapiens*	*H. sapiens*
Tissue	Placenta (decidual tissues)	Uterus (endometrial tissues)	Uterus (endometrial tissues)
Samples in RSA group	3	5	24
Samples in control group	5	5	24
Reference	PMID: 36439123	PMID: 22025212	PMID: 36369952

Abbreviations: GEO, Gene Expression Omnibus; RSA, recurrent spontaneous abortion.

We collected 1452 metabolic reprogramming‐related genes (MRRGs) from the GeneCards database [15] (https://www.genecards.org/) using the keyword “metabolic reprogramming”, retaining only those MRRGs classified as “protein coding” with a relevance score > 1. Additionally, we identified 5 MRRGs from PubMed literature [16], resulting in a total of 1457 MRRGs after merging and removing duplicates, as shown in Supporting Information S1.

For the two GEO datasets GSE26787 and GSE165004, probe‐to‐gene symbol mapping was performed using the platform‐specific annotation files (GPL570 for GSE26787, GPL16699 for GSE165004). When a probe corresponded to multiple gene symbols, the first valid non‐OTTHU/OTTHUMG gene symbol was preferentially extracted. When multiple probes mapped to the same gene, the average expression value of these probes was taken as the final expression level for that gene. After probe annotation, the two datasets were merged based on common genes. Batch effects between the two datasets were then corrected using the ComBat function from the sva package (version 3.50.0) [17]. Specifically, a batch vector was defined to distinguish samples from GSE26787 and GSE165004, and all other parameters were left at their default values. Following batch correction, the expression matrix was normalised using the normalizeBetweenArrays function from the limma package (version 3.58.1) [18] to reduce inter‐sample distribution differences. The effectiveness of batch correction and normalisation was evaluated by principal component analysis (PCA) [19] on the expression matrices before and after processing, generating an integrated dataset of 29 RSA and 29 control samples.

### 2.2 Differentially Expressed Genes (DEGs) Associated With Metabolic Reprogramming in RSA

Based on the sample grouping of the integrated GEO dataset, differential expression analysis of genes was conducted using R package limma (version 3.58.1) [18], with DEGs identified by |logFC| > 0.5 and *p* < 0.05. Up‐regulated DEGs had logFC > 0.5, while down‐regulated DEGs had logFC < −0.5. The Benjamini‐Hochberg (BH) method was used for *p* value correction, and results were visualised with a volcano plot from the R package ggplot2 (version 3.4.4).

To identify metabolic reprogramming‐related differentially expressed genes (MRRDEGs) associated with RSA, all DEGs with |logFC| > 0.5 and *p* < 0.05 from the integrated GEO datasets were intersected with MRRGs, illustrated by a Venn diagram. A heatmap of MRRDEGs was created using the R package pheatmap (version 1.0.12).

### 2.3 Weighted Gene Co‐Expression Network Analysis (WGCNA)

WGCNA was performed using the R package WGCNA package [20] (version 1.72–5) to identify phenotype‐associated gene modules. Prior to network construction, the expression variance of each gene across all samples was calculated. Genes were ranked by variance from highest to lowest, and only the top 70% of genes with the highest variance were retained for subsequent analysis. Subsequently, a co‐expression network was built based on these highly variable genes. The soft‐thresholding power (*β*) was selected as 18 according to the scale‐free topology fit index. An adjacency matrix was then built using this *β*, and the topological overlap matrix (TOM) was calculated. Hierarchical clustering was performed on the TOM‐based dissimilarity matrix. Dynamic tree cutting was applied to identify co‐expression modules, with a minimum module size of 200 and a merge cut height of 0.2. The correlation between modules and the RSA phenotype was evaluated using correlation analysis. To determine an appropriate threshold for identifying phenotype‐associated modules, we compared the module retention results under different correlation cutoffs. Considering the correlation coefficient magnitude, statistical significance, and the number of modules retained, we ultimately selected |*r*| > 0.30 and *p* < 0.05 as the screening criteria to define candidate modules associated with the RSA phenotype for subsequent analyses. Genes from these significant modules were then intersected with MRRDEGs using Venn analysis to identify core module genes.

### 2.4 Construction of Diagnostic Model for RSA

To identify a robust set of diagnostic features from candidate module genes, a multistage modelling strategy was adopted. Logistic regression was first employed to preliminarily assess the association between candidate genes and the disease phenotype, retaining features significantly associated with RSA to ensure statistical relevance and interpretability. Subsequently, Support Vector Machine Recursive Feature Elimination (SVM‐RFE) was applied to further refine the gene combination, leveraging its ability to optimise classification performance and minimise the error rate. Finally, Least Absolute Shrinkage and Selection Operator (LASSO) regression was employed to further compress variables, reduce redundancy and construct a relatively parsimonious diagnostic model. LASSO shrinks coefficients and removes irrelevant features to minimise overfitting. This sequential workflow was designed to balance feature selection, model stability and interpretability.

Univariate logistic regression analysis was performed using the R function glm, with sample group as the dependent variable (Control = 0, RSA = 1) and the expression levels of the intersecting genes between WGCNA module genes and MRRDEGs as independent variables. A two‐sided *p* < 0.05 was used as the preliminary screening criterion to identify genes significantly associated with RSA. Regression coefficients, odds ratios (ORs), and their 95% confidence intervals (CIs) were calculated, and the results were visualised as a forest plot using the forestplot package.

Subsequently, diagnostic feature selection was performed using the multiple SVM‐RFE algorithm [21]. In the recursive elimination stage, a linear kernel C‐classification SVM (cost = 10, cachesize = 500, scale = FALSE) was used to rank features under 5‐fold cross‐validation (CV). When remaining features > 50, the set was halved per iteration, otherwise, features were eliminated one by one. To determine the optimal feature number, each top‐ranked subset was evaluated with a radial basis function kernel, and hyperparameters (gamma ranging from 2^−12^ to 2° and cost ranging from 2^−6^–2^6^) were tuned via grid search under 5‐fold CV. The optimal feature number was finally determined by identifying the point that minimised the average classification error rate and maximised the accuracy on the validation curve.

Finally, LASSO regression was performed using the glmnet package [22] (version 4.1–8) with family = “binomial” and alpha = 1. The optimal penalty parameter λ was selected via 10‐fold CV using the cv.glmnet function. The classification error rate was used as the evaluation metric, and the genes with non‐zero coefficients corresponding to λ.min were selected as the final model genes. The risk score was calculated based on the expression values of these genes and their LASSO regression coefficients as follows:

$$\text{Risk}\;\text{Score}=\sum_{i}\text{Coefficient}\;\left({\text{gene}}_{i}\right)*\text{mRNA}\;\text{Expression}\;\left({\text{gene}}_{i}\right)$$

### 2.5 Validation of the Diagnostic Model for RSA

The R package pROC (version 1.18.5) was used to plot the ROC curve and calculate the area under the curve (AUC) to assess the diagnostic effectiveness of the risk score for RSA. A nomogram [23] was constructed based on the four LASSO‐selected genes using logistic regression with the R package rms (version 6.7–1) to illustrate the interrelations and contributions of the model genes. Calibration analysis was conducted to create a calibration curve based on the results of LASSO regression, evaluating the accuracy and discriminative ability of the RSA diagnostic model. Additionally, decision curve analysis (DCA) [24] was performed employing the R package ggDCA (version 1.1) to generate decision curves based on the model genes in the integrated GEO dataset.

### 2.6 Protein‐Protein Interaction (PPI) Network

The STRING [25] (https://string‐db.org/) database is a resource for searching known and predicted protein‐protein interactions. In this study, we utilised the STRING database to construct a PPI network related to the model genes based on a minimum interaction score greater than 0.40. We also used the GeneMANIA online tool [26] (https://genemania.org/) to predict functionally similar genes for the model genes.

### 2.7 Construction of Regulatory Networks

We analysed the regulatory effects of transcription factors (TFs) on model genes using TFs retrieved from the ChIPBase database [27] (http://rna.sysu.edu.cn/chipbase/) and visualised the mRNA‐TF regulatory network using Cytoscape software [28].

RNA‐binding protein (RBP) [29] plays a crucial role in gene regulation and is essential for various biological activities, including RNA synthesis, alternative splicing, modification, transport, and translation. Based on the StarBase v3.0 database (https://starbase.sysu.edu.cn/), we predicted the target RBPs for the model genes and visualised the mRNA‐RBP regulatory network using Cytoscape software.

### 2.8 Gene Ontology (GO) and Pathway Enrichment Analysis

GO analysis [30] is a commonly used method for large‐scale functional enrichment studies, encompassing biological processes (BP), cellular components (CC), and molecular functions (MF). The Kyoto Encyclopaedia of Genes and Genomes (KEGG) [31] is a widely used database that stores information about genomes, biological pathways, diseases, and drugs. We utilised the R package clusterProfiler [32] (version 4.10.0) to perform GO and KEGG enrichment analyses for the model genes, applying the filtering criteria of *p* < 0.05 and an FDR value (*q* value) < 0.25.

### 2.9 Gene Set Enrichment Analysis (GSEA)

GSEA [33] is used to assess gene distribution trends in ranked lists linked to phenotypes, evaluating their contributions. We ranked genes from the integrated GEO dataset by logFC values between RSA and control groups, then used the R package clusterProfiler [32] (version 4.10.0) for GSEA on all genes. The parameters used for the GSEA are as follows: seed value of 2020, a minimum of 10 genes and a maximum of 500 genes per gene set. The gene set c2.cp.all.v2022.1.Hs.symbols.gmt [All Canonical Pathways] (3050) was retrieved from the Molecular Signatures Database (MSigDB) [34] for the enrichment analysis. The selection criteria for the GSEA included a *p* < 0.05 and an FDR value (*q* value) < 0.25, with the *p* value adjustment method being BH.

### 2.10 Gene Set Variation Analysis (GSVA)

GSVA [35] transforms the gene expression matrix across different samples into pathway‐level activity scores, enabling evaluation of biological pathway enrichment across samples. In this study, we obtained the gene set c2.cp.v2023.2.Hs.symbols.gmt from the MSigDB and employed the R package GSVA (version 1.50.0) to perform GSVA on all genes in the integrated GEO dataset. We calculated the functional enrichment differences between the RSA and control group with a significance threshold of *p* < 0.05 and BH adjustment.

### 2.11 Immune Infiltration Analysis

Single sample gene set enrichment analysis (ssGSEA) [36] quantifies the relative abundance of immune cell infiltration in samples by labelling various immune cell subtypes and calculating enrichment scores to create an infiltration matrix. Correlations among immune cells were analysed using the Spearman algorithm, visualised with a heatmap using the R package pheatmap (version 1.0.12). Additionally, correlations between model genes and immune cells were computed and visualised in a bubble chart using R package ggplot2 (version 3.4.4) for significant results (*p* < 0.05).

Cell identity by estimating relative subsets of RNA transcripts (CIBERSORT) [37] uses linear support vector regression to deconvolute transcriptomic data, estimating immune cell composition in mixed populations. Using the CIBERSORT algorithm in conjunction with the LM22 signature gene matrix, we filtered for immune cell enrichment scores greater than zero, ultimately obtaining a comprehensive immune cell infiltration matrix from the integrated GEO dataset. The results were visualised using a bar chart to display the proportions of different immune cell types. We used the Spearman algorithm to calculate correlations among immune cells and created a heatmap with R package pheatmap (version 1.0.12). We also computed correlations between model genes and immune cells, retaining *p* < 0.05, and visualised these results with a bubble chart using R package ggplot2 (version 3.4.4).

### 2.12 Quality Control of Single‐Cell Dataset

The single‐cell RNA‐seq dataset GSE214607, comprising 3 RSA and 5 control samples, was downloaded from GEO. The CreateSeuratObject function in the Seurat package (version 5.0.1) [38] was used to import the count matrix. Cells were retained if they expressed at least 200 genes, and genes were retained if detected in at least 3 cells. Quality control was performed by filtering out cells with RNA counts < 500, feature counts < 250, log_10_ (Genes per UMI) < 0.8, or mitochondrial gene content > 20%. Data were then normalised using the NormalizeData function with the default LogNormalize method. The top 2000 highly variable genes were identified using the FindVariableFeatures function with the vst method. The data were scaled using ScaleData to remove sequencing depth effects. PCA was performed, and the elbow plot was used to determine the number of significant principal components (PCs). The first 30 PCs were selected for downstream analysis. Because only one single‐cell dataset was included in this study, no cross‐dataset integration or batch correction was performed. The K‐nearest neighbour graph was constructed using the FindNeighbors function with the first 30 PCs. Clusters were identified using the FindClusters function with a resolution of 0.6 (determined using the clustree package). Uniform manifold approximation and projection (UMAP) was applied for dimensionality reduction and visualisation using the RunUMAP function.

### 2.13 Cell Type Annotation and Single Cell Differentially Expressed Genes (scDEGs)

First, we performed cell type annotation on the RSA single‐cell dataset GSE214607 using the SingleR function from the R package SingleR [39] (version 2.4.1), referencing the ImmGenData dataset to identify cell types. Subsequently, we employed the DotPlot function to display the expression levels of model genes across different cell types.

To identify differential genes between cell clusters, we utilised the FindAllMarkers function, comparing gene expression in each cell with that of all other cells through the Wilcoxon rank‐sum test. We retained the top 10 differential genes for each cell cluster as our scDEGs for further analysis.

### 2.14 Human Tissues

This study included six women with RSA, aged 25–35 years at gestational week (GW) 6–12, from whom decidual samples were obtained. Participants were recruited from the Obstetrics and Gynaecology Department of the International Peace Maternity and Child Health Hospital affiliated with Shanghai Jiao Tong University School of Medicine during January 2020 to August 2022. Women with RSA were excluded if they had uterine/cervical malformations, endocrine/immunologic/metabolic diseases, abnormal parental or abortus karyotypes, exposure to toxins/infectious agents, or comorbidities like hypertension, diabetes, or thyroid dysfunction. Six age‐matched healthy controls (HCs, 25–35 years) undergoing voluntary termination of normal, unwanted pregnancies at GW 6–12 were also recruited. HCs had no history of spontaneous abortion, preeclampsia, intrauterine growth retardation, or preterm birth. The Medical Ethics Committee of the aforementioned hospital approved the protocol, which complied with the Declaration of Helsinki. All subjects provided written informed consent before participation.

### 2.15 Cell Culture

HTR‐8/SVneo cells (HTR‐8), derived from first‐trimester extravillous trophoblasts, were kindly provided by Dr. PK Lala (University of Western Ontario, London, Ontario, Canada). Cell culture utilised DMEM/F12 medium (Gibco, Thermo Fisher Scientific, Waltham, MA, USA) enriched with 10% foetal bovine serum (FBS, Gibco). The telomerase‐immortalised human endometrial stromal cells (T‐HESC) (ATCC, CRL‐4003, USA) were cultured in DMEM/F12 medium supplemented with 10% FBS (Gibco).

### 2.16 Plasmids Transfection and T‐HESC Decidualization

To induce overexpression, pcDNA 3.1 vector was inserted with the open reading frame of SREBF2 or the negative control vector (NC) were used for transfection (GenePharma, Shanghai, China). The plasmids were transfected into the cells using Lipofectamine 3000 Reagent (Invitrogen, Thermo Fisher Scientific) and reduced‐serum medium (Opti‐MEM, Gibco) according to the manufacturer's protocol.

To induce in vitro decidualization, T‐HESC cells were treated for 4 days with a differentiation medium containing medroxyprogesterone acetate (MPA, 10 μM, Selleck Chemicals, Houston, TX, USA) and 8‐Br‐cAMP (0.5 mM, Selleck Chemicals) in serum‐free conditions. The medium was refreshed every 48 h. Decidualization was evaluated by measuring the expression levels of two classical markers: prolactin (PRL) and insulin‐like growth factor binding protein 1 (IGFBP‐1). For conditioned medium preparation, the culture supernatant was collected after transfection and stimulus treatments, replaced with fresh medium, and then incubated for another 24 h. The resulting conditioned medium (T‐HESC CM) was used to assess trophoblast cell migration.

### 2.17 Quantitative RT‐PCR

RNA was extracted using TRIzol reagent (Invitrogen). Concentration and purity of the isolated RNA were measured with a NanoDrop 2000c spectrophotometer (Thermo Fisher Scientific). Complementary DNA (cDNA) was synthesised using the PrimeScript RT reagent Kit (Perfect Real Time) (Takara Bio, Shiga, Japan). Quantitative PCR was carried out with TB Green Premix Ex Taq II (Tli RNaseH Plus) (Takara Bio) on a real‐time PCR system. The ΔCt value for each target gene was calculated by subtracting the Ct value of the endogenous control GAPDH. Relative mRNA expression levels were determined using the 2^(−ΔΔCt) method. The primers used are listed in Supporting Information S2: Table S1.

### 2.18 Western Blot

Cells were lysed using RIPA buffer (Thermo Fisher Scientific) supplemented with protease and phosphatase inhibitors. Equal amounts of protein were separated by SDS‐PAGE and transferred onto PVDF membranes. After blocking with 5% non‐fat milk, the membranes were incubated overnight at 4°C with primary antibodies against SREBF2 (Abcam, ab30682, 126 kDa, Cambridge, UK) and GAPDH (Abcam, ab8245, 36 kDa). Following TBST washes, the membranes were incubated with HRP‐conjugated secondary antibodies. Protein bands were visualised using Western HRP Substrate (Millipore, Burlington, MA, USA) and imaged with an Amersham Imager 600 system (GE Healthcare, Uppsala, Sweden).

### 2.19 CCK‐8 Assay

Cell viability was assessed using a Cell Counting Kit‐8 (CCK‐8) assay (Yeasen Biotechnology, Shanghai, China). Briefly, cells were seeded in 96‐well plates at a density of 2 × 10^3^ cells per well and cultured for 0, 12, 24, 48, or 72 h. Following the incubation period, CCK‐8 solution was added to each well. Absorbance was measured at 450 nm using a microplate reader (BioTek, Winooski, VT, USA).

### 2.20 Transwell Migration Assay

A suspension of HTR‐8 cells (200 μL) in serum‐free DMEM/F12 was seeded into the upper chamber of a transwell insert (8 μm pore size, Corning, New York, NY, USA) at a density of 5 × 10^4^ cells per well. The lower chambers received 600 μL of conditioned medium. After 48 h of incubation at 37°C, the inserts were washed with ice‐cold PBS, fixed with 4% paraformaldehyde, and stained with crystal violet. Non‐migratory cells remaining on the upper membrane surface were gently removed using a cotton swab. Following air‐drying, the number of invaded cells in each treatment group was quantified under a microscope (Leica Microsystems, Wetzlar, Germany) and normalised relative to the control.

### 2.21 Statistical Analysis

All data processing and statistical analyses in this study were performed using R software (version 4.3.0). Unless otherwise specified, comparisons between two groups of continuous variables were assessed using the independent Student's *t*‐test for normally distributed variables and the Mann‐Whitney *U* test (Wilcoxon rank‐sum test) for non‐normally distributed variables. The Kruskal‐Wallis test was employed for comparisons involving three or more groups. Correlations between molecules were quantified through Spearman's correlation analysis. All statistical tests were two‐tailed, with *p* < 0.05 considered statistically significant unless specifically indicated.

## 3 Results

### 3.1 DEGs Associated With Metabolic Reprogramming in RSA

**FIGURE 1:**
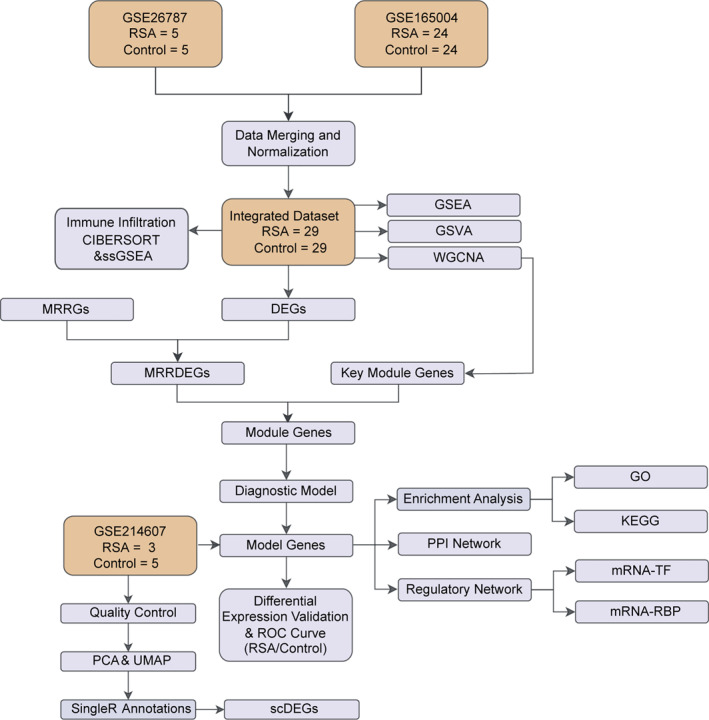
The analysis workflow of our study. DEGs, differentially expressed genes; GO, Gene Ontology; GSEA, gene set enrichment analysis; GSVA, gene set variation analysis; KEGG, Kyoto Encyclopaedia of Genes and Genomes; MRRDEGs, metabolic reprogramming‐related differentially expressed genes; MRRGs, metabolic reprogramming‐related genes; PCA, principal component analysis; PPI, protein‐protein interaction; RBP, RNA‐binding protein; ROC, receiver operating characteristic; RSA, recurrent spontaneous abortion; scDEGs, single‐cell differentially expressed genes; ssGSEA, single sample gene set enrichment analysis; TF, transcription factor; UMAP, Uniform Manifold Approximation and Projection; WGCNA, weighted gene co‐expression network analysis.

The analysis workflow is illustrated in Figure 1. Batch effects between datasets GSE26787 and GSE165004 were removed, resulting in an integrated GEO dataset. PCA plots were used to visualise the low‐dimensional feature distributions before and after batch correction (Supporting Information S2: Figure S1A and S1B). The results confirmed that batch effects were effectively minimised after the removal process.

**FIGURE 2:**
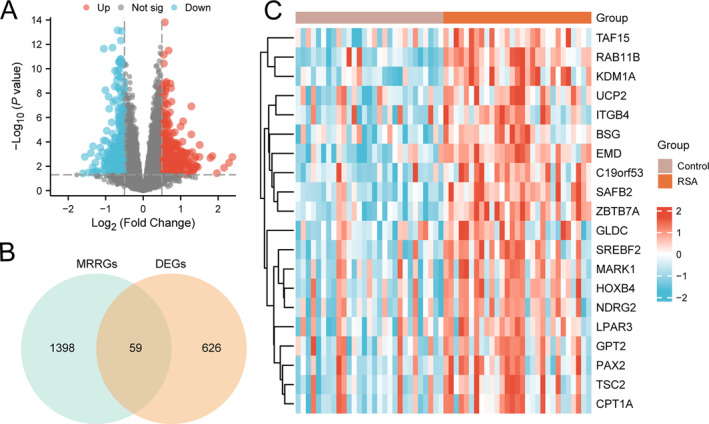
DEGs associated with metabolic reprogramming in RSA. (A) The volcano plot of DEGs between the RSA group and the control group in the integrated GEO dataset. (B) The Venn diagram of DEGs and MRRGs in the integrated GEO dataset. (C) The heatmap of the top 20 MRRDEGs in the integrated GEO dataset. RSA group in orange and control group in light brown. The heat map shows high expression in red and low expression in blue.DEGs, differentially expressed genes; MRRDEGs, metabolic reprogramming‐related differentially expressed genes; MRRGs, metabolic reprogramming‐related genes; RSA, recurrent spontaneous abortion.

The integrated GEO dataset was divided into an RSA group and a control group. Differential gene expression analysis between these groups was conducted, which identified 685 DEGs meeting the thresholds of |logFC| > 0.5 and *p* < 0.05. Among these, 374 genes were upregulated and 311 were downregulated. These results are visualised in the volcano plot shown in Figure 2A.

To identify MRRDEGs, we intersected all DEGs meeting the criteria (|logFC| > 0.5 and *p* < 0.05) with MRRGs, as shown in the Venn diagram (Figure 2B). This yielded 59 MRRDEGs, with detailed information provided in Supporting Information S1. Based on these overlapping genes, we analysed the expression differences of MRRDEGs among different sample groups in the integrated GEO dataset and visualised the top 20 MRRDEGs using a heatmap (Figure 2C).

### 3.2 WGCNA

**FIGURE 3:**
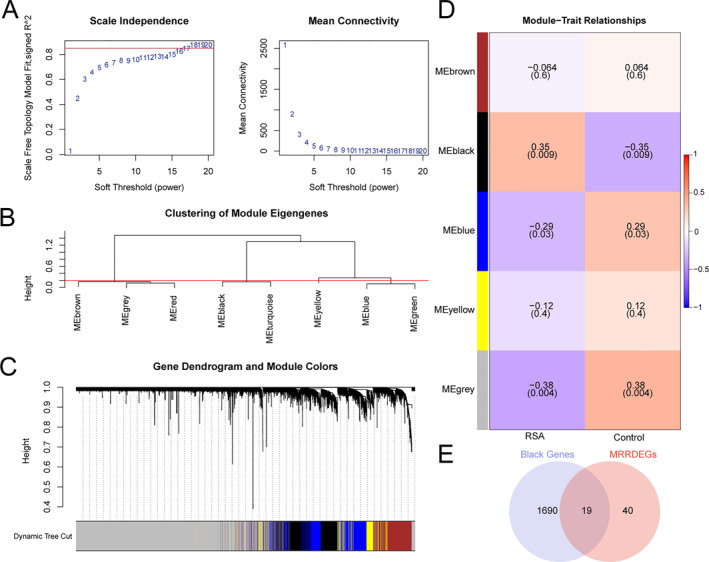
WGCNA (A) The scale‐free topology fit index (left panel) and mean connectivity (right panel) are plotted against different soft‐thresholding powers. (B) The results of module clustering for the top 70% of variance genes are presented. (C) The clustering results for the top 70% of variance genes are shown, with the upper part representing the hierarchical clustering dendrogram and the lower part depicting gene modules. (D) The correlation analysis results between the clustering modules of the top 70% variance genes and the RSA group and control group are displayed. The absolute value of the correlation coefficient (*r* value) below 0.3 is considered weak or uncorrelated, while a value between 0.3 and 0.5 is regarded as weakly correlated. Red indicates positive correlation, while blue represents negative correlation. (E) A Venn diagram showing the MRRDEGs and the genes included in the MEblack module. MRRDEGs, metabolic reprogramming‐related differentially expressed genes; RSA, recurrent spontaneous abortion; WGCNA, weighted gene co‐expression network analysis.

WGCNA was performed to identify co‐expression modules from the integrated GEO datasets. The scale‐free topology fit index was evaluated across different soft threshold powers (Figure 3A), and a soft threshold of 18 was selected as the optimal value, achieving a fit index of 0.85. Using this threshold, hierarchical clustering was applied to identify co‐expression modules from the top 70% most variable genes (Figure 3B). At a merge cut‐off of 0.2, five modules were identified: MEbrown, MEblack, MEblue, MEyellow, and MEgrey. The relationships between genes and modules are visualised in Figure 3C. Module‐trait correlations were evaluated based on gene expression profiles (Figure 3D). Based on the criteria |*r*| > 0.30 and *p* < 0.05, the MEblack module was selected for further analysis. The intersection between the 59 MRRDEGs and genes in the MEblack module yielded 19 overlapping genes (Figure 3E), listed as follows: *C19orf53, CPT1A, FHL2, GPT2, GPX2, HMGCS2, IDH1, ITGB4, PAX2, PPARG, PRRX1, SLC38A5, SQLE, SREBF2, THBS1, TLR2, TRPM6, TSC2, UCP2*.

### 3.3 Construction of a Diagnostic Model for RSA

**FIGURE 4:**
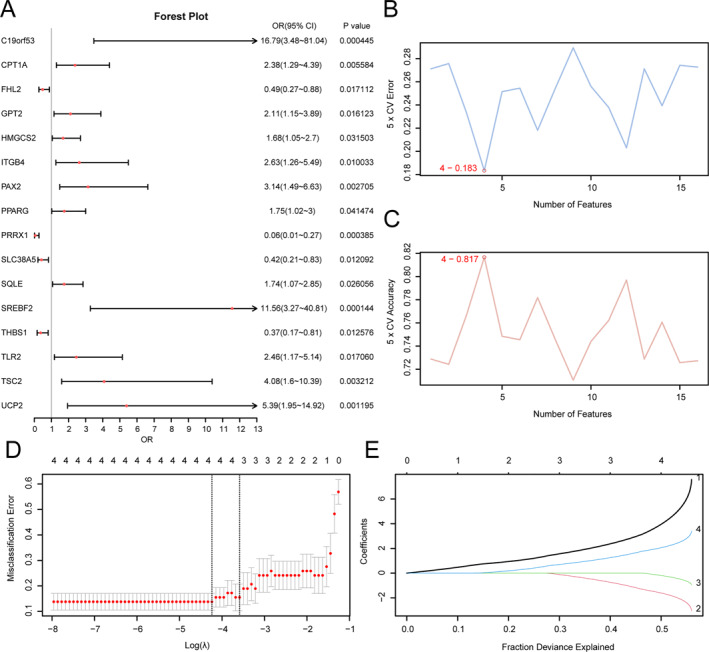
Construction of a diagnostic model for RSA. (A) Forest plot showing the 16 statistically significant module genes included in the logistic regression model for the diagnosis of RSA. (B) and (C) Visualisation of the number of genes with the lowest error rate (B) and the highest accuracy (C) obtained from the SVM‐RFE algorithm. (D) and (E) The diagnostic model plot (D) and variable trajectory plot (E) of the LASSO regression model. LASSO, Least Absolute Shrinkage and Selection Operator. RSA, recurrent spontaneous abortion; SVM‐RFE, Support Vector Machine Recursive Feature Elimination.

To evaluate the diagnostic potential of the 19 module genes for RSA, a logistic regression model was constructed and visualised using a forest plot (Figure 4A). Sixteen genes showed statistical significance (*p* < 0.05) in the model. Subsequently, an SVM‐RFE model was built using these genes, and the optimal number of genes yielding the lowest error rate (Figure 4B) and highest accuracy (Figure 4C) was identified. The SVM‐RFE model achieved peak accuracy when incorporating four genes.

These four genes were further analysed using LASSO regression to develop a diagnostic model for RSA. The LASSO model and variable trajectory are shown in Figure 4D,E. The final model included four genes: *SREBF2* (sterol regulatory element‐binding protein 2), *PPARG* (peroxisome proliferator‐activated receptor gamma), *SQLE* (squalene epoxidase), and *UCP2* (uncoupling protein 2). Based on the regression coefficients from the LASSO analysis, a risk score was calculated using the following formula:

RiskScore=SREBF2∗(4.444)+PPARG∗(−1.79)+SQLE∗(−0.307)+UCP2∗(2.233)

### 3.4 Validation of the Diagnostic Model for RSA

**FIGURE 5:**
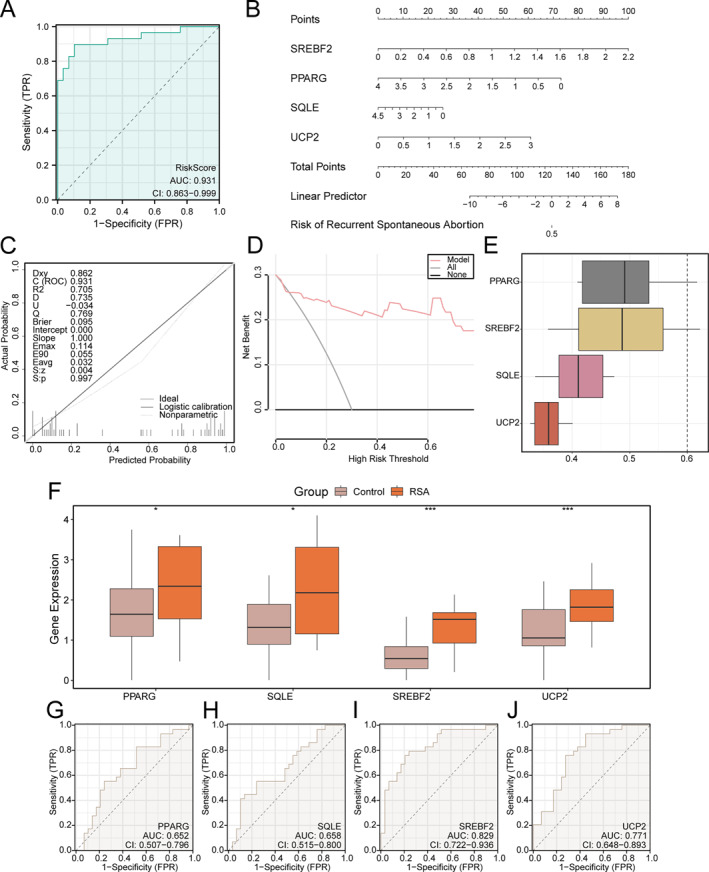
Validation of the diagnostic model and model genes for RSA. (A) The ROC curve of the risk score in the integrated GEO dataset. (B) The nomogram for model genes in the RSA diagnostic model based on the integrated GEO dataset. (C) and (D) The calibration curve (C) and DCA (D) for the RSA diagnostic model based on model genes. In the DCA plot, the vertical axis represents net benefit, while the horizontal axis represents threshold probability. (E) Boxplot results of Friends analysis for model genes. (F) Model genes in the integrated GEO dataset are presented through a group comparison plot for the RSA group and the control group. In the group comparison plot, light brown represents control samples, while orange represents RSA samples. *p < 0.05, ***p < 0.001. (G)–(J) The ROC curves for model genes *PPARG* (G), *SQLE* (H), *SREBF2* (I), and *UCP2* (J) in the integrated GEO dataset are shown. An AUC greater than 0.5 suggests that the expression of the molecule is associated with a trend towards event occurrence, with the closer the AUC is to 1, the better the diagnostic effectiveness. An AUC value between 0.5 and 0.7 suggests low accuracy, while an AUC between 0.7 and 0.9 indicates moderate accuracy, and an AUC above 0.9 reflects high accuracy. AUC, area under the curve; DCA, decision curve analysis; FPR, false positive rate; ROC, receiver operating characteristic; RSA, recurrent spontaneous abortion; TPR, true positive rate.

An ROC curve was generated based on the risk score to evaluate the model's performance (Figure 5A). To assess the robustness and potential overfitting of the diagnostic model, we performed internal validation using bootstrap resampling (1000 iterations) (Supporting Information S2: Figure S2). The model demonstrated excellent discriminative ability with an AUC of 0.931. Furthermore, the bootstrap validation yielded a 95% CI of 0.8537–0.9881, indicating high stability and reliability of the diagnostic model in distinguishing RSA from control samples. To further evaluate the diagnostic model, a nomogram was constructed using the model genes to illustrate their contributions within the integrated GEO dataset (Figure 5B). The results reveal that *SREBF2* has the highest predictive utility in the RSA diagnostic model. The calibration curve was used to assess the agreement between predicted probabilities and actual outcomes (Figure 5C). The dashed calibration line shows slight deviation from the ideal diagonal, indicating modest variation from perfect prediction. DCA was performed to examine the clinical net benefit of the diagnostic model (Figure 5D). The model's curve remains well above the “All positive” and “All negative” lines within a certain range, demonstrating substantial clinical utility. Finally, functional similarity (Friends) analysis was conducted to evaluate the biological relevance of the model genes (Figure 5E). The results indicate that *SREBF2* plays a crucial role in RSA and is the gene closest to the cut‐off value (cut‐off value = 0.6).

### 3.5 Validation of the Expression and Diagnostic Performance of the Model Genes

To evaluate the expression of model genes in the integrated GEO dataset, a group comparison plot was generated to display their differential expression between RSA and control samples (Figure 5F). The analysis revealed highly significant upregulation of *SREBF2* and *UCP2* in RSA samples (*p* < 0.001), while *PPARG* and *SQLE* also showed significant differential expression (*p* < 0.05). ROC curves were subsequently plotted to assess the diagnostic performance of these genes (Figure 5G–J). The expression levels of *PPARG* and *SQLE* exhibited limited classification accuracy (0.5 < AUC < 0.7), whereas *SREBF2* and *UCP2* demonstrated moderate discriminatory ability in distinguishing RSA from control samples (0.7 < AUC < 0.9).

**FIGURE 6:**
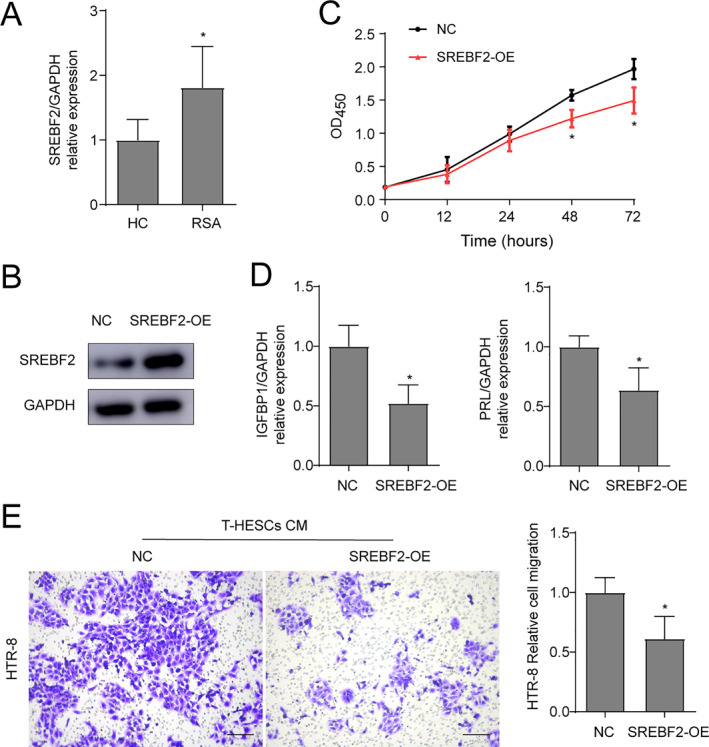
*SREBF2* overexpression impairs decidualization and trophoblast migration in vitro. (A) Expression levels of *SREBF2* in decidual tissues from women with RSA and healthy controls (HCs) were detected by qPCR. (B) T‐HESCs were transfected with negative control (NC) or SREBF2‐overexpression vector (SREBF2‐OE), and overexpression efficiency was confirmed by western blot. (C) CCK‐8 assay showed that *SREBF2* overexpression inhibited T‐HESC proliferation. (D) After transfection with NC or SREBF2‐OE vectors, T‐HESCs were decidualized with 8‐Br‐cAMP and MPA. The mRNA expression of decidual markers PRL and IGFBP‐1 was measured by qPCR. (E) Conditioned media (CM) from decidualized T‐HESCs, which had been transfected with NC or SREBF2‐OE vectors, were collected and applied to HTR‐8 trophoblast cells to induce migration, which was assessed using a transwell assay. *p < 0.05. Data are expressed as the mean ± SD. Scale bars represent 100 μm.

We also validated the expression of the four model genes in decidual tissues from women with RSA and healthy controls using qPCR (Supporting Information S2: Figure S3; Figure 6A). In line with the bioinformatic predictions, *SREBF2, UCP2* and *SQLE* were upregulated in RSA decidua compared to controls. For *PPARG*, our qPCR did not detect a significant difference.

### 3.6 *SREBF2* Overexpression Impairs Decidualization and Trophoblast Migration in Vitro

Given that *SREBF2* demonstrated the highest utility in the diagnostic model and a significant role in the biological processes of RSA, we further investigated its functions in RSA. Quantitative PCR analysis revealed that the mRNA expression of *SREBF2* was significantly increased in the decidual tissue obtained from the RSA group compared to that from the HCs (Figure 6A). We transfected T‐HESC cells with either a NC vector or an SREBF2‐overexpression (SREBF2‐OE) vector. The transfection efficiency was confirmed by western blot (Figure 6B). A CCK‐8 assay demonstrated that enforced expression of SREBF2 significantly slowed down the proliferation of T‐HESCs compared to the NC group (Figure 6C). Furthermore, we induced decidualization in the transfected T‐HESCs using a combination of 8‐Br‐cAMP and MPA and analysed the expression levels of the classic decidual markers PRL and IGFBP‐1. The expression of both PRL and IGFBP‐1 was profoundly attenuated in cells overexpressing SREBF2, indicating that SREBF2 overexpression inhibits the decidualization process of human endometrial stromal cells (Figure 6D). Given the critical role of decidual cells in mediating trophoblast function, we collected conditioned media (CM) from T‐HESCs transfected with NC or SREBF2‐OE vectors after decidualization induction. We then evaluated the effect of this CM on the migratory capacity of human trophoblast cells (HTR‐8). Transwell migration assays showed that the CM from SREBF2‐overexpressing T‐HESCs significantly reduced the migration of HTR‐8 cells compared to the CM from the NC group (Figure 6E).

### 3.7 Construction of PPI Network and Regulatory Network of Model Genes

**FIGURE 7:**
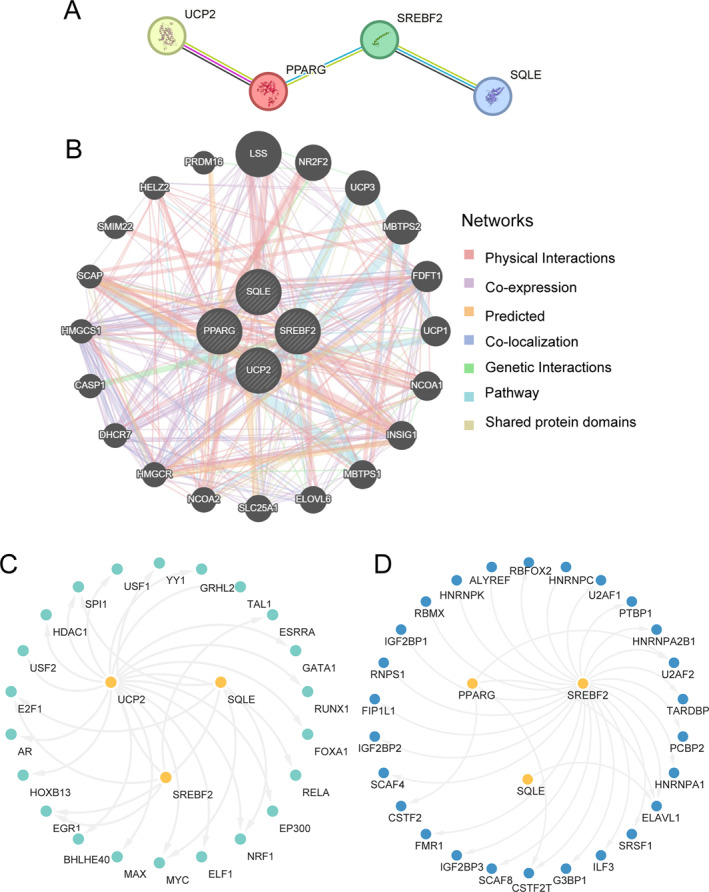
Construction of PPI network and regulatory network of model genes (A) The PPI network of model genes, calculated using the STRING database. (B) The interaction network of functionally similar genes predicted for the four model genes, obtained from the GeneMANIA website. In the figure, the circles represent the model genes and the functionally similar genes that are associated with them, with the line colours indicating the types of functional relationships. (C) The mRNA‐TF regulatory network of model genes. (D) The mRNA‐RBP regulatory network of model genes. In the figures, yellow represents mRNA, green represents TF, and blue represents RBP. PPI, protein‐protein interaction; RBP, RNA‐binding protein; TF, transcription factor.

A PPI network was constructed for the four model genes (*UCP2*, *PPARG*, *SREBF2*, and *SQLE*) (Figure 7A). The results demonstrate measurable interactions among all four genes. The interaction network of these genes and their functionally associated partners was further predicted (Figure 7B). The network comprises the four model genes and 20 functionally similar genes.

To identify regulatory factors associated with the model genes, TFs and RBPs targeting them were predicted. The resulting mRNA‐TF regulatory network comprises three model genes and 22 TFs (Figure 7C, details in Supporting Information S1). Similarly, the mRNA‐RBP interaction network includes three model genes and 26 RBPs (Figure 7D, details in Supporting Information S1).

### 3.8 GO and KEGG Enrichment Analysis

To further investigate the functional relevance of the four model genes in RSA, GO and KEGG enrichment analyses were performed. The results revealed significant enrichments across multiple categories (Supporting Information S2: Table S2).

**FIGURE 8:**
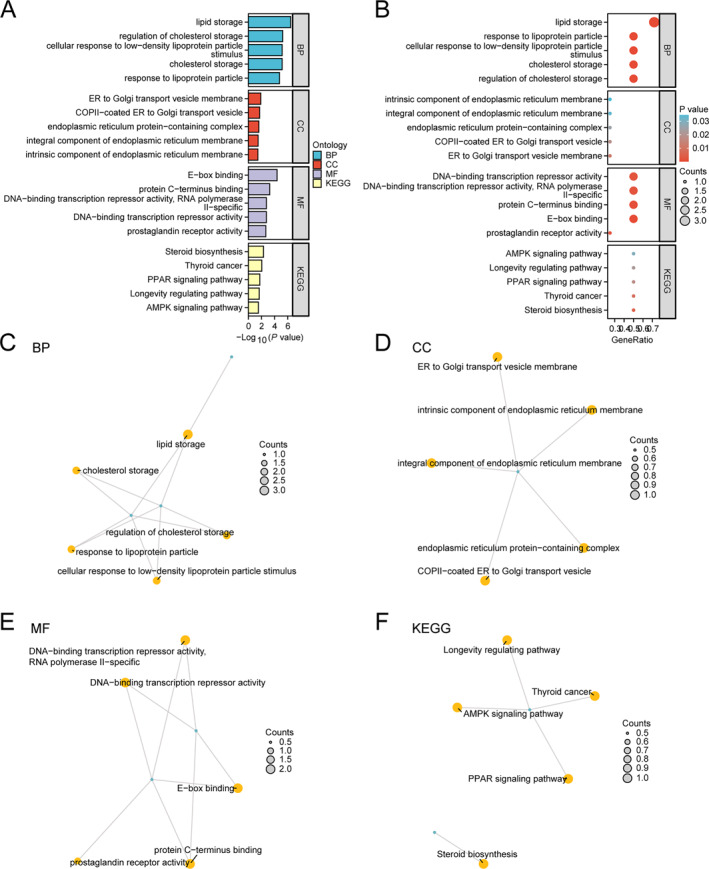
GO and KEGG enrichment analysis. (A) Bar chart presenting the results of GO and KEGG enrichment analysis for model genes, showcasing BP, CC, MF, and KEGG. (B) Bubble chart illustrating the results of GO and KEGG enrichment analysis for model genes, displaying BP, CC, MF, and KEGG. The vertical axis represents GO terms and KEGG terms. In the bubble chart, the size of the bubbles represents the number of genes, and the colour of the bubbles indicates the size of the *p* value, with redder colours indicating smaller *p* values and bluer colours indicating larger *p* values. (C)–(F) Network diagrams showing the results of GO and KEGG enrichment analysis for model genes: BP (C), CC (D), MF (E), and KEGG (F). Yellow nodes represent entries, blue nodes represent molecules, and the connecting lines represent the relationships between entries and molecules. BP, biological process; CC, cellular component; GO, Gene Ontology; KEGG, Kyoto Encyclopaedia of Genes and Genomes; MF, molecular function.

Biologically, the genes were primarily involved in processes such as lipid storage, regulation of cholesterol storage, and cellular response to low‐density lipoprotein stimuli. For cellular components, enrichments included the ER‐to‐Golgi transport vesicle membrane, COPII‐coated vesicles, and endoplasmic reticulum protein‐containing complexes. Molecular functions featured E‐box binding, protein C‐terminus binding, and DNA‐binding transcription repressor activity. KEGG pathway analysis indicated involvement in steroid biosynthesis, the PPAR signalling pathway, thyroid cancer, longevity regulation, and the AMPK signalling pathway. The results are summarised visually using bar charts and bubble plots (Figure 8A,B). Network diagrams for BP, CC, MF, and KEGG terms were generated based on the enrichment analyses (Figure 8C–F). Larger nodes indicate a greater number of molecules associated with that term.

### 3.9 GSEA

**FIGURE 9:**
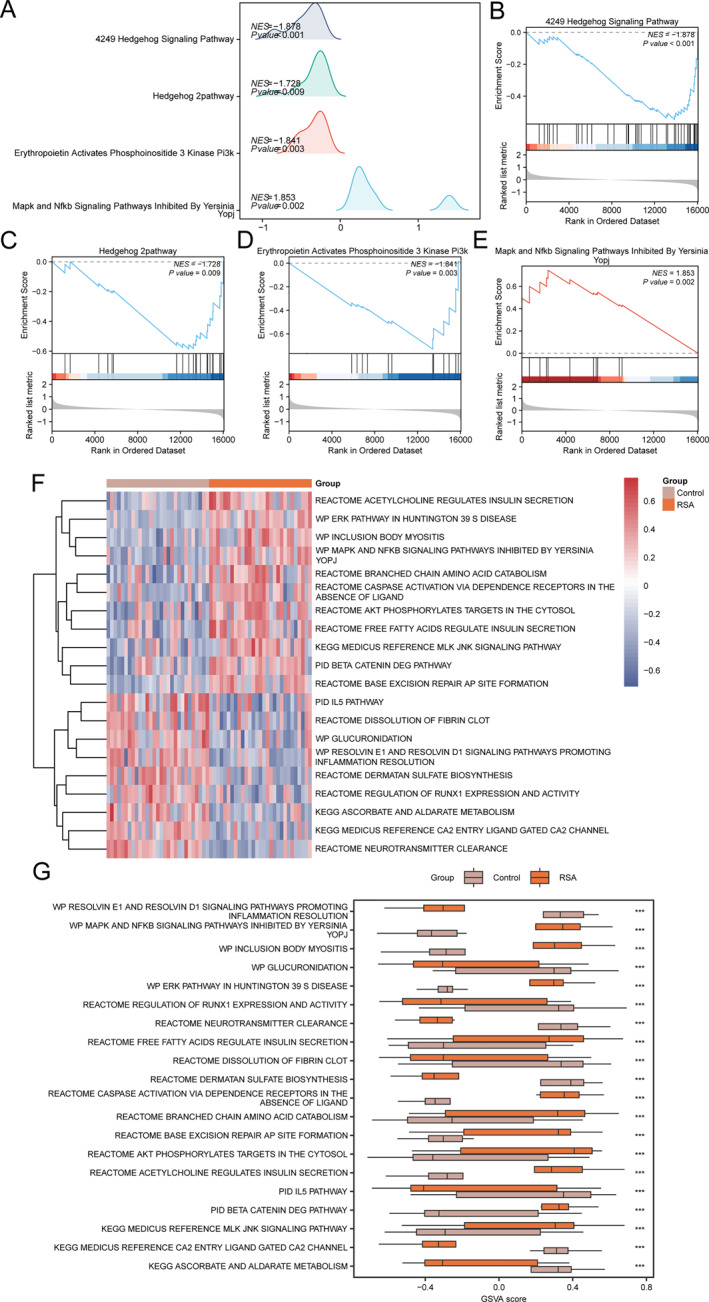
GSEA and GSVA. (A) The mountain plot illustrates the results of GSEA in the integrated GEO datasets. (B)–(E). The GSEA indicates that the integrated GEO datasets are significantly enriched in the 4249 Hedgehog signalling pathway (B), Hedgehog 2 pathway (C), erythropoietin activates phosphoinositide 3 kinase (PI3K) (D), and the MAPK and NF‐κB signalling pathways inhibited by *Yersinia* Yopj (E). (F) and (G). The results of GSVA in the integrated GEO dataset showing the heatmap (F) and group comparison chart (G) between the RSA group and the control group. Orange represents the RSA group, while light brown represents the control group. ***p < 0.001. In the heatmap, blue indicates low enrichment, while red indicates high enrichment. GSEA, gene set enrichment analysis; GSVA, gene set variation analysis; RSA, recurrent spontaneous abortion.

GSEA results, visualised using a mountain plot (Figure 9A, details in Supporting Information S2: Table S3), revealed significant enrichment of genes involved in key pathways. These included the 4249 Hedgehog signalling pathway, Hedgehog 2 pathway, erythropoietin‐activated phosphoinositide 3‐kinase (PI3K) pathway, and MAPK and NF‐κB signalling pathways inhibited by *Yersinia* YopJ (Figure 9B–E), among other biologically relevant functions.

### 3.10 GSVA

GSVA was performed on the integrated GEO dataset to compare pathway activity between the RSA and control groups (details in Supporting Information S2: Table S4). The top 20 most differentially expressed pathways (*p* < 0.05), ranked by absolute logFC values, are shown in a heatmap (Figure 9F). Differential activity was further validated using the Mann‐Whitney *U* test and displayed in a grouped comparison plot (Figure 9G). Significantly altered pathways (*p* < 0.05) included Reactome neurotransmitter clearance, KEGG Medicus reference Ca^2+^ entry ligand‐gated Ca^2+^ channel, KEGG ascorbate and aldarate metabolism, Reactome regulation of RUNX1 expression and activity, Reactome dermatan sulphate biosynthesis, WP resolvin E1 and resolvin D1 signalling pathways promoting inflammation resolution, WP glucuronidation, Reactome dissolution of fibrin clot, PID IL5 pathway, Reactome base excision repair AP site formation, PID beta‐catenin deg pathway, KEGG Medicus reference MLK JNK signalling pathway, Reactome free fatty acids regulate insulin secretion, Reactome AKT phosphorylates targets in the cytosol, Reactome caspase activation via dependence receptors in the absence of ligand, Reactome branched‐chain amino acid catabolism, WP MAPK and NF‐κB signalling pathways inhibited by *Yersinia* YopJ, WP inclusion body myositis, WP ERK pathway in Huntington's disease, and Reactome acetylcholine regulates insulin secretion.

### 3.11 Immune Infiltration Analysis by ssGSEA Algorithm

**FIGURE 10:**
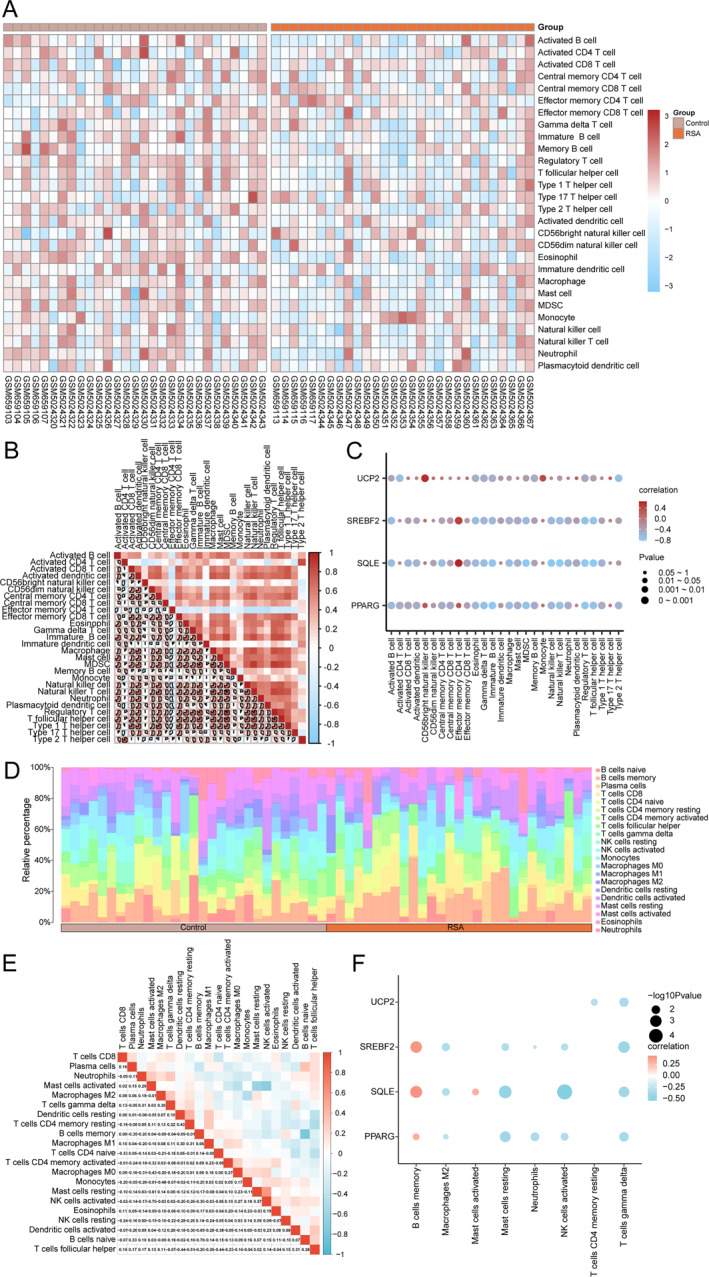
Immune infiltration analysis by ssGSEA and CIBERSORT algorithm. (A) Heatmap of immune cell infiltration levels between the RSA and the control group by ssGSEA algorithm. (B) Correlation heatmap of immune cells by ssGSEA algorithm. (C) Correlation bubble chart of model genes and the infiltration levels of immune cell types by ssGSEA algorithm. (D) Bar plot of immune cell proportions between the RSA and the control group by CIBERSORT algorithm. (E) Heatmap of the correlation of immune cells by CIBERSORT algorithm. (F) Bubble map of correlation between model genes and immune cell infiltration proportions by CIBERSORT algorithm. The absolute value of the correlation coefficient (*r* value) is categorised as weak or uncorrelated below 0.3, weakly correlated between 0.3 and 0.5, moderately correlated between 0.5 and 0.8, and strongly correlated above 0.8. Orange represents the RSA group, while light brown represents the control group. Red indicates positive correlation, and blue indicates negative correlation. The intensity of colour represents the strength of the correlation.

The ssGSEA algorithm was applied to the integrated GEO expression matrix to quantify the infiltration abundance of 28 immune cell types. A heatmap was generated to visualise differences in immune cell infiltration between groups (Figure 10A). Correlation analysis among these immune cells revealed that myeloid‐derived suppressor cells (MDSC) and natural killer T cells (NKT) showed the strongest positive correlation (*r* = 0.899), while plasmacytoid dendritic cells (pDC) and effector memory CD4^+^ T cells exhibited the highest negative correlation (*r* = −0.592) (Figure 10B).

Further correlation analysis between the four model genes and immune cells was visualised in a bubble chart (Figure 10C). *SQLE* correlated most positively with effector memory CD4^+^ T cells (*r* = 0.637, *p* < 0.05) and most negatively with NKT cells (*r* = −0.824, *p* < 0.05). *UCP2* showed the strongest positive correlation with CD56^bright^ natural killer cells (*r* = 0.584, *p* < 0.05) and a strong negative correlation with activated CD4^+^ T cells (*r* = −0.685, *p* < 0.05). *PPARG* was weakly positively correlated with effector memory CD4^+^ T cells (*r* = 0.278, *p* < 0.05) but highly negatively correlated with immature B cells (*r* = −0.761, *p* < 0.05). *SREBF2* demonstrated moderate positive correlation with effector memory CD4^+^ T cells (*r* = 0.442, *p* < 0.05) and a strong negative correlation with T follicular helper cells (*r* = −0.711, *p* < 0.05).

### 3.12 Immune Infiltration Analysis by CIBERSORT Algorithm

Using the CIBERSORT algorithm, the infiltration levels of 22 immune cell types were assessed in the integrated GEO dataset. The relative proportions of these cells are shown in a bar chart (Figure 10D). A correlation heatmap of immune cell infiltration revealed the strongest positive correlation between resting mast cells and activated natural killer cells (NK cells) (*r* = 0.572), and the strongest negative correlation between naïve B cells and memory B cells (*r* = −0.764) (Figure 10E)

Correlations between model genes and immune infiltration are displayed in a bubble chart (Figure 10F). *SQLE* was positively correlated with memory B cells (*r* = 0.433, *p* < 0.05) and negatively correlated with activated NK cells (*r* = −0.535, *p* < 0.05). *PPARG* showed a positive correlation with memory B cells (*r* = 0.298, *p* < 0.05) and a negative correlation with resting mast cells (*r* = −0.398, *p* < 0.05). *UCP2* was negatively correlated with gamma delta T cells (*r* = −0.354, *p* < 0.05). *SREBF2* correlated positively with memory B cells (*r* = 0.422, *p* < 0.05) and negatively with gamma delta T cells (*r* = −0.423, *p* < 0.05).

### 3.13 Single‐Cell RNA Sequencing Analysis

**FIGURE 11:**
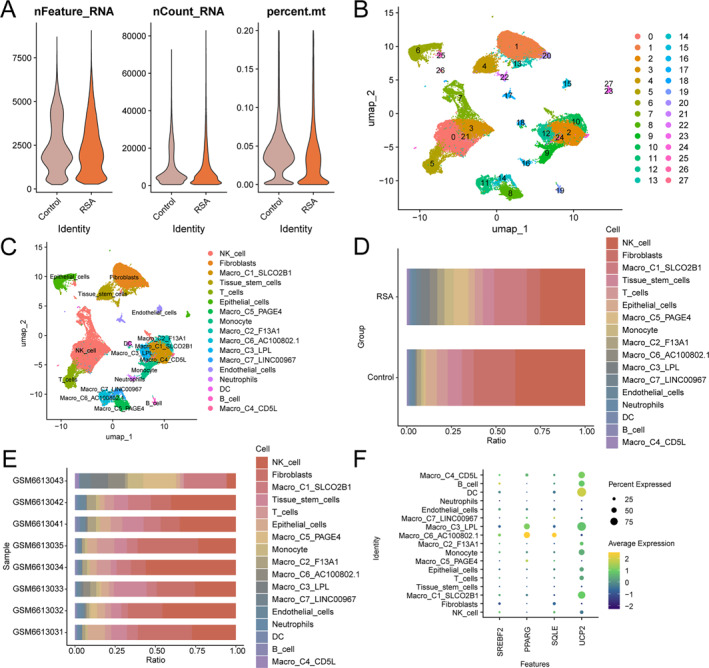
Single‐cell RNA sequencing analysis. (A) Violin plot displays the distributions of nFeature_RNA, nCount_RNA, and log_10_ (Genes per UMI) per cell in the single‐cell dataset GSE214607. (B) 66,387 cells clustered into 28 cell clusters through UMAP. (C) Cell annotation into 17 cell types: natural killer cells (NK Cells), fibroblasts, macrophages with high SLCO2B1 expression (Macro_C1_SLCO2B1), macrophages with high F13A1 expression (Macro_C2_F13A1), macrophages with high LPL expression (Macro_C3_LPL), macrophages with high CD5L expression (Macro_C4_CD5L), macrophages with high PAGE4 expression (Macro_C5_PAGE4), macrophages with high AC100802.1 expression (Macro_C6_AC100802.1), macrophages with high LINC00967 expression (Macro_C7_LINC00967), tissue stem cells, T cells, epithelial cells, monocytes, endothelial cells, neutrophils, dendritic cells (DCs), and B cells. (D) Bar chart showing cell proportions in RSA samples and control samples. (E) Bar chart showing cell proportions in individual samples from the RSA group and control group. (F) Bubble chart visualising the expression levels of four model genes in the single‐cell dataset. The yellower the colour, the higher the expression level, the bluer the colour, the lower the expression level, the larger the circle, the higher the expression proportion of the gene within the cell group. RSA, recurrent spontaneous abortion; UMAP, Uniform Manifold Approximation and Projection.

The single‐cell dataset GSE214607, comprising 3 RSA and 5 control samples, was processed for analysis. Genes detected in fewer than 3 cells and cells expressing fewer than 200 genes were excluded. A violin plot (Figure 11A) shows the distributions of nFeature_RNA, nCount_RNA, and log_10_ (Genes per UMI) per cell. Quality control filtering was then applied resulting in 66,387 high‐quality cells. Dimensionality reduction and visualisation via UMAP identified 28 distinct cell clusters (Figure 11B).

Cell types across the 28 clusters were annotated, identifying 17 distinct populations (Figure 11C), including NK cells, fibroblasts, macrophages with high SLCO2B1 expression (Macro_C1_SLCO2B1), macrophages with high F13A1 expression (Macro_C2_F13A1), macrophages with high LPL expression (Macro_C3_LPL), macrophages with high CD5L expression (Macro_C4_CD5L), macrophages with high PAGE4 expression (Macro_C5_PAGE4), macrophages with high AC100802.1 expression (Macro_C6_AC100802.1), macrophages with high LINC00967 expression (Macro_C7_LINC00967), tissue stem cells, T cells, epithelial cells, monocytes, endothelial cells, neutrophils, dendritic cells (DCs), and B cells.

Bar charts illustrating cell type proportions revealed notable differences between RSA and control samples (Figure 11D), as well as across individual samples (Figure 11E). Expression patterns of the four model genes within the single‐cell data are shown in a bubble plot (Figure 11F). *UCP2* was enriched in DCs, B cells, monocytes, Macro_C1_SLCO2B1, Macro_C3_LPL, Macro_C4_CD5L, T cells, and epithelial cells. *SQLE* showed primary enrichment in Macro_C6_AC100802.1, while *PPARG* was mainly expressed in Macro_C3_LPL and Macro_C6_AC100802.1.

### 3.14 DEGs Analysis in Single Cell Group

**FIGURE 12:**
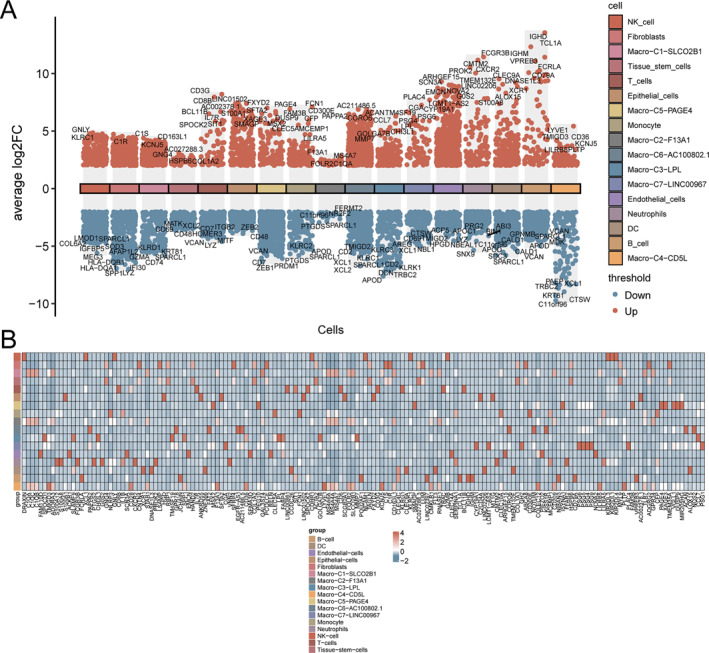
DEGs analysis in single‐cell group. (A) Volcano plot of differential genes in cells. Red indicates genes that are upregulated in the cells, while blue indicates genes that are downregulated in the cells. (B) Heatmap of scDEGs. In the heatmap, red represents high expression, and blue represents low expression. scDEG, single‐cell differentially expressed genes.

DEGs across cell types were identified with thresholds set at |logFC| > 2.00 and adj.*p* < 0.05. The results were visualised with a volcano plot (Figure 12A). Expression level of the top 10 upregulated genes is shown in a heatmap (Figure 12B) and Table 2.

**TABLE 2:** DEGs analysis in single‐cell group.

Cell type	Top 10 upregulated genes
NK cells	KLRC1, GZMB, NCAM1, XCL1, KIR2DL4, CDHR1, KIR2DL3, GNLY, KIR2DL1, DRAXIN
Fibroblasts	C1S, PCOLCE, C1R, LUM, SFRP4, CNTN4, ABCA8, ADGRG2, TMEM35A, LSAMP
Macro_C1_SLCO2B1	SLCO2B1, STAB1, GAL3ST4, CMKLR1, TLR7, LILRB5, ADORA3, CD209, KCNJ5, CD163L1
Macro_C2_F13A1	C1QA, C1QB, C1QC, RNASE1, MS4A7, MS4A6A, FOLR2, F13A1, MS4A4A, GPR34
Macro_C3_LPL	LPL, TM4SF19, SPP1, CHI3L1, FABP4, SPOCD1, MMP7, CCL7, SLAMF9, GOLGA7B
Macro_C4_CD5L	LYVE1, COLEC12, LILRB5, CD36, F13A1, KCNJ5, CD28, SPP1, PLTP, TMIGD3
Macro_C5_PAGE4	PAGE4, XAGE3, PEG10, VGLL1, ISYNA1, DUSP9, SMAGP, FAM3B, MSX2, MIR205HG
Macro_C6_AC100802.1	NOTUM, PAPPA2, CORO6, GPR78, FAT2, AC211486.5, KISS1R, ACAN, NOS2, EGFR‐AS1
Macro_C7_LINC00967	CGA, PSG5, PSG9, CYP19A1, PSG1, PSG4, PLAC4, PSG6, LCMT1‐AS2, GH2
Tissue stem cells	RBP1, MYL9, TAGLN, HAND2, HSPB6, COL1A1, COL1A2, AC027288.3, ACTA2, GNG4
T cells	TRAC, CD3G, BCL11B, SPOCK2, IL7R, LINC00861, CD6, SIT1, CD8B, KLRG1
Epithelial cells	LINC01502, FXYD2, S100A1, MT1G, C2CD4A, SCGB2A1, AC002378.1, SFTA2, ANKRD55, HGD
Monocytes	SERPINA1, FCN1, CD300E, IL1B, CFP, LILRA5, NLRP3, CLEC5A, CLEC10A, MCEMP1
Endothelial cells	CLEC14A, EMCN, ACKR1, RELN, NOVA2, FAM110D, PIEZO2, ARHGEF15, SEMA3D, SCN3A
Neutrophils	S100A8, FCGR3B, G0S2, CMTM2, PTGS2, CXCR2, PROK2, S100A12, FFAR2, VNN2
DCs	DNASE1L3, CLEC9A, XCR1, FLT3, ZNF366, LINC02206, CCDC26, ALOX15, CLEC4C, TMEM132E
B cells	CD79A, IGHM, VPREB3, IGHD, JCHAIN, LINC02397, NIBAN3, FCRLA, TCL1A, POU2AF1

## 4 Discussion

RSA results in recurrent embryo or foetal death, infertility, and poses significant challenges to women's health, with profound implications for their emotional and physical well‐being. Currently, the lack of effective diagnostic tools and treatments highlights the urgent need for innovative management strategies. Our study integrates multiple GEO datasets to elucidate the molecular mechanisms associated with RSA, suggesting a lipid metabolism‐centred metabolic remodelling feature in RSA. We aim to improve both the understanding and management of this complex disorder.

Our analysis identified a total of 685 DEGs within the integrated GEO dataset, with 374 upregulated and 311 downregulated genes. Among these, 59 genes were linked to metabolic reprogramming. These findings suggest a substantial role for metabolic reprogramming in RSA, laying the groundwork for further exploration into its pathological mechanisms. Among these, four key genes (*SREBF2*, *PPARG*, *SQLE* and *UCP2*) were highlighted as core components of a diagnostic model for RSA.

*SREBF2* has been identified as a key regulator of lipid metabolism and cholesterol homoeostasis, suggesting its potential role in altering the maternal lipid environment and thereby affecting embryo implantation and development [40]. Furthermore, its involvement in inflammatory processes may further underscore its relevance in RSA, given the established link between chronic inflammation and impaired reproductive outcomes [41]. In our diagnostic model, *SREBF2* was assigned the highest risk coefficient and exhibited a more prominent predictive contribution, therefore it was selected as a representative core gene for further functional validation. In this study, we demonstrate the functional significance of *SREBF2* in RSA pathogenesis. *SREBF2* expression is significantly upregulated in decidual tissues from women with RSA. In vitro overexpression of *SREBF2* in human endometrial stromal cells (T‐HESCs) inhibits cell proliferation and disrupts decidualization, as indicated by reduced expression of the decidual markers PRL and IGFBP‐1. The compromised decidualization caused by *SREBF2* overexpression creates a hostile microenvironment that attenuates the migratory capacity of trophoblasts, a process essential for successful embryo implantation and placentation.

In addition to *SREBF2*, other genes including *PPARG*, *SQLE*, and *UCP2* also contribute to related pathways implicated in RSA. *PPARG* modulates inflammatory and metabolic processes, with dysregulation potentially leading to insulin resistance and chronic inflammation that may adversely affect pregnancy [42, 43]. *SQLE*, a rate‐limiting enzyme in cholesterol synthesis, influences steroidogenesis and cellular differentiation, with possible implications for hormonal balance and immune modulation in miscarriage [44, 45, 46]. *UCP2* participates in mitochondrial function and oxidative stress response, and may also contribute to immune regulation relevant to maternal‐foetal tolerance [47, 48]. We verified through PCR experiments that *SREBF2*, *SQLE* and *UCP2* are upregulated in decidual samples from RSA, while *PPARG* shows no statistically significant difference between the RSA group and the control group. This inconsistency may arise from differences in sample size, or technical platform. Notably, our single‐cell data revealed that *PPARG* expression is restricted to a specific macrophage subset (Macro_C3_LPL and Macro_C6_AC100802.1) rather than being ubiquitous. Therefore, bulk tissue analyses may overestimate or underestimate cell‐type‐specific changes. Future validation of *PPARG* should employ cell‐type‐resolved methods such as flow cytometry or single‐cell qPCR.

Our diagnostic model demonstrated high predictive accuracy (AUC > 0.9). Although *SREBF2* showed the highest coefficient and single‐gene AUC, the full four‐gene model outperformed any single‐gene model, suggesting that the diagnostic performance arises from the combined effect of the four genes rather than from *SREBF2* alone. Meanwhile, *PPARG*, *SQLE* and *UCP2* also demonstrated RSA‐related biological features in differential expression, model selection, functional enrichment, immune infiltration correlations and single‐cell expression distribution, indicating that they also contribute to model construction. Thus, the four‐gene model is best understood as a joint molecular framework, within which *SREBF2* may play a relatively key role. In addition, exploring the model's applicability in associated gestational complications, such as preeclampsia, may elucidate shared pathogenic mechanisms underlying placental dysfunction. Clinical adoption of this predictive framework may represent a paradigm shift in RSA management, transitioning from reactive symptom‐based care to proactive risk‐stratified management strategies. This advance holds significant potential for improving maternal‐foetal outcomes and reducing the healthcare burden associated with recurrent pregnancy loss.

GO and KEGG enrichment analyses of the model genes reveal a coherent theme centred on lipid metabolic regulation. The significant enrichment in biological processes such as “lipid storage” and “regulation of cholesterol storage” underscores their functionally interconnected in lipid metabolism and cholesterol homoeostasis. Thus, we propose that a lipid metabolism‐centred metabolic remodelling feature exists at the maternal‐foetal interface in RSA. The enrichment of *PPARG* in the “AMPK signalling pathway” and “longevity regulating pathway” suggests the induction of adaptive, pro‐survival metabolic responses [49]. On the other hand, the molecular function term “DNA‐binding transcription repressor activity” shared by *SREBF2* and *PPARG* may indicate concurrent repressive signalling that could constrain cellular processes under metabolic stress. Thus, the four model genes are not biologically independent of each other and may jointly participate in metabolic imbalance and immune microenvironment remodelling at the maternal‐foetal interface.

GSEA further revealed suppression of Hedgehog signalling, essential for embryogenesis and placental development, alongside enrichment of *Yersinia* YopJ‐mediated inhibition of MAPK/NFκB pathways at the maternal‐foetal interface in RSA [50, 51]. This combination implies concurrent developmental impairment and compromised protective immunity at the maternal‐foetal interface, which may synergistically undermine pregnancy establishment and maintenance. The detection of a bacterial immune evasion signature also raises the possibility of subclinical infection, dysbiosis, or autoimmune mimicry as potential etiological factor.

GSVA highlighted broad dysregulation in RSA, including defects in inflammation resolution, metabolic homoeostasis, neuroendocrine regulation, and stress response. Together, these findings indicate a systemic failure to integrate immune tolerance, metabolic adaptation, and survival signals in early gestation of RSA. These insights suggest several therapeutic directions: ER‐related complexes could serve as diagnostic biomarkers, resolvin analogues may offer new anti‐inflammatory strategies, and AMPK/insulin‐based subtyping could facilitate early metabolic intervention [52, 53].

Analysis of the immune microenvironment in RSA by ssGSEA revealed a significant positive correlation between MDSCs and NKT cells. MDSCs, which suppress T‐cell responses and promote immunosuppression, likely engage in bidirectional crosstalk with NKT cells [54, 55]. Under physiological conditions, MDSCs may polarise NKT cells toward tolerogenic phenotypes, while NKT cells enhance MDSCs recruitment via cytokines. In RSA, dysregulated MDSCs function could disrupt this balance, leading to pro‐inflammatory NKT activation and contributing to placental dysfunction [56, 57, 58]. This hypothesis requires further experimental validation. Conversely, pDCs correlated negatively with effector memory CD4^+^ T cells. Although pDCs support foetal tolerance by inducing regulatory T cells (Tregs) and modulating inflammation via interferon signalling [59], the role of effector memory CD4^+^ T cells in RSA remains unclear. Hypothetically, effector memory CD4^+^ T cells may drive RSA pathogenesis through pro‐inflammatory mechanisms [60]. Notably, model genes *SQLE*, *PPARG* and *SREBF2* positively correlated with effector memory CD4^+^ T cells in ssGSEA analysis. Further research is warranted to explore the mechanistic underpinnings of these relationships and their implications for immunotherapy strategies.

CIBERSORT analysis showed a strong positive correlation between resting mast cells and activated NK cells, and a negative correlation between naïve and memory B cells. The role of resting mast cells in RSA remains undefined, though their interaction with NK cells may involve cytokine‐mediated priming of cytotoxic or inflammatory responses at the maternal‐foetal interface [61, 62]. Additionally, single‐cell approaches are needed to determine if “resting” mast cells exhibit pregnancy‐specific regulatory features. Memory B cell expansion, inversely linked to naïve B cell depletion, may reflect chronic antigen exposure or dysregulated differentiation [63]. The model genes *SQLE*, *PPARG* and *SREBF2* all demonstrated a positive correlation with memory B cell. The potential role of memory B cells in RSA remains largely unexplored. However, based on their established functions in immune memory and autoimmunity, memory B cells might contribute to RSA through aberrant antibody production or by sustaining chronic inflammation at the maternal‐foetal interface [64]. These cells could also interact with T follicular helper cells or DCs to amplify pro‐inflammatory responses, potentially disrupting placental immune tolerance [65].

The single‐cell differential gene analysis revealed distinct transcriptional signatures across diverse cell populations, highlighting the molecular heterogeneity of the tissue microenvironment. Cytotoxic effector genes (KLRC1, GZMB, GNLY) dominated NK cells, while T and B cells expressed lineage‐defining receptors (TRAC/CD3G; CD79A/IGHM) [66, 67, 68]. Monocytes exhibited pro‐inflammatory profiles (IL1B, NLRP3), neutrophils displayed antimicrobial effectors (S100A8/S100A12), and DCs upregulated antigen‐presentation genes (CLEC9A, XCR1) [69, 70, 71]. Notably, macrophage subsets exhibited significant heterogeneity, defined by unique marker genes (SLCO2B1, F13A1, LPL, CD5L, PAGE4, AC100802.1, LINC00967). The specific expression of these markers implies specialised functions for each subset. Further supporting this heterogeneity, model genes *SQLE* and *PPARG* were selectively enriched in macrophage subsets (Macro_C6_AC100802.1 and Macro_C3_LPL, respectively), implicating the metabolic‐immune axis shaping macrophage functionality. Macro_C6_AC100802.1 may represent a previously unrecognised population with dual roles in cholesterol synthesis and lipid storage [72]. Previous studies indicating a conflicting role for *PPARG* activation in macrophage differentiation [73]. *PPARG* likely drives lipid accumulation and increases inflammatory response in Macro_C3_LPL [74]. The broad enrichment of *UCP2* in DCs, B cells, monocytes, and multiple macrophage subsets (Macro_C1_SLCO2B1, C3_LPL, C4_CD5L) implies its importance in immune adaptation. According to previous research, *UCP2* may reprograms M1 macrophages into M2 phenotypes by promoting oxidative phosphorylation [75, 76]. Similarly, its presence in epithelial cells and T cells underscores its importance in maintaining cellular homoeostasis across diverse microenvironments [77, 78]. Future studies incorporating multi‐omic approaches, such as epigenomic, proteomic, and spatial transcriptomic profiling, will be essential to elucidate how transcriptional diversity translates into functional phenotypes. Such insights could ultimately facilitate precise targeting of macrophage subpopulations for therapeutic intervention in RSA.

Several limitations should be acknowledged. First, we adopted a stepwise modelling strategy to balance feature selection, interpretability, and parsimony, rather than to systematically benchmark different algorithms. Thus, we did not compare the sequential workflow with models using logistic regression, SVM‐RFE, or LASSO alone. While such comparison could provide additional insight, it was beyond the primary scope of this study, which aimed to construct a clinically interpretable diagnostic signature through a biologically informed stepwise pipeline. Future studies with larger, independent cohorts should benchmark multiple algorithms against our model to improve predictive performance and generalisability. Second, the relatively small sample size and multi‐step feature selection strategy may increase the risk of overfitting. Consequently, external validation remains a key priority. At present, the high degree of heterogeneity across public RSA datasets, stemming from differences in tissue types and technical platforms, prevented us from performing independent validation. When larger, more consistent independent datasets become available, we will further validate the model's robustness and generalisability. Third, important clinical variables, such as metabolic status, body mass index (BMI), lipid profiles, gestational age, as well as ultrasound parameters and immune profiling, were not fully considered. These factors are known to influence lipid metabolism and pregnancy outcomes, and their absence may affect the model's clinical applicability and predictive performance. Future studies should recruit larger, independent cohorts of RSA patients and healthy controls with detailed phenotyping and assess the model's performance across different clinical subgroups. Fourth, functional validation focused primarily on *SREBF2*. Therefore, the relative contributions of the four genes and their molecular mechanisms require further elucidation using independent cohorts and experimental studies. Future work will systematically characterise the functional roles of *UCP2*, *SQLE*, and *PPARG* using in vitro and in vivo models.

Although the present study relies on traditional machine learning methods, deep learning has recently emerged as a powerful tool for transcriptomic biomarker discovery [79, 80, 81, 82, 83, 84]. These approaches capture complex, non‐linear relationships in high‐dimensional omics data through attention mechanisms, multi‐scale feature fusion, generative cross‐modal integration and hyperbolic graph learning. Notably, deep learning models can jointly integrate multi‐omics data and model hierarchical or scale‐free network structures. These capabilities are highly relevant to RSA, which involves intricate crosstalk among metabolic reprogramming, immune tolerance and stromal‐trophoblast interactions. Deep learning could potentially integrate transcriptomic data with clinical variables or other omics layers to build more robust and interpretable models. Future studies should explore these advanced computational strategies to further elucidate the molecular mechanisms of RSA and facilitate clinical translation.

In summary, our study suggests the presence of a lipid metabolism‐centred metabolic remodelling feature in RSA. Integrative analysis of bulk and single‐cell RNA sequencing datasets has identified core MRRDEGs in RSA and established a machine learning‐based diagnostic model with strong discriminatory capacity. In the context of current understanding of RSA pathogenesis, we highlight three interconnected axes: decidual dysfunction, trophoblast impairment and disruption of maternal‐foetal immune tolerance. Our results show that *SREBF2* upregulation in RSA decidua suppresses endometrial stromal cell proliferation and decidualization, contributing to decidual dysfunction. In turn, this compromised decidual environment attenuates trophoblast migration, linking decidual defects to trophoblast insufficiency. Meanwhile, immune infiltration and single‐cell analyses reveal that the model genes are enriched in specific macrophage subsets and correlate with memory B cells and effector memory CD4^+^ T cells, suggesting that lipid‐centric metabolic remodelling may also regulate immune intolerance at the maternal‐foetal interface. Thus, our work advances the concept that RSA involves a lipid‐centric metabolic remodelling that may simultaneously impacts decidualization, trophoblast function and immune adaptation.

Emerging evidence suggests that RSA should not be viewed in isolation. Instead, it may be an early clinical manifestation of a spectrum of adverse pregnancy outcomes that share common pathogenic roots. A growing body of epidemiological and biological data indicates a significant overlap between RSA and other major obstetric syndromes, most notably preeclampsia, foetal growth restriction, and spontaneous preterm birth [85]. Subsequent validation with larger clinical cohorts will be essential to refine these tools and improve the management of RSA as well as other pregnancy‐related diseases, ultimately leading to better pregnancy outcomes.

## Author Contributions

**Fan Wu:** methodology, software, data curation, investigation, writing – original draft. **Chuanmei Qin:** software, investigation, validation. **Xiaowei Wei:** data curation. **Qian Li:** data curation. **Yi Yuan:** software, validation. **Weihong Zeng:** supervision, writing – review and editing. **Yi Lin:** conceptualization, supervision, funding acquisition, writing – review and editing.

## Funding

This research was funded by the National Key Research and Development Programme of China (2018YFC1002800), the National Natural Science Foundation of China (82471722, 82471726, 82171669 and 81971403), the Shanghai Jiao Tong University Trans‐Med Awards Research (Major Project) (20210201), and the Funds for Outstanding Newcomers, Shanghai Sixth People's Hospital (X‐3664).

## Conflicts of Interest

The authors declare no conflicts of interest.

## Supporting information


Supporting Information S1

Supporting Information S2

## Data Availability

We analyzed data from the publicly accessible Gene Expression Omnibus (GEO) database (https://www.ncbi.nlm.nih.gov/geo/).
