# Supplementary material for: Metabolic Reprogramming in Recurrent Spontaneous Abortion: Key Biomarkers Identification and Diagnostic Model Development
Source: IET Syst Biol. 2026 Jun 17;20(1):e70078. doi: 10.1049/syb2.70078 (PMC13275172; doi:10.1049/syb2.70078)
Supplement: Supplementary file 2 — Supporting Information S2 [file SYB2-20-e70078-s002.pdf]

Supplementary Information

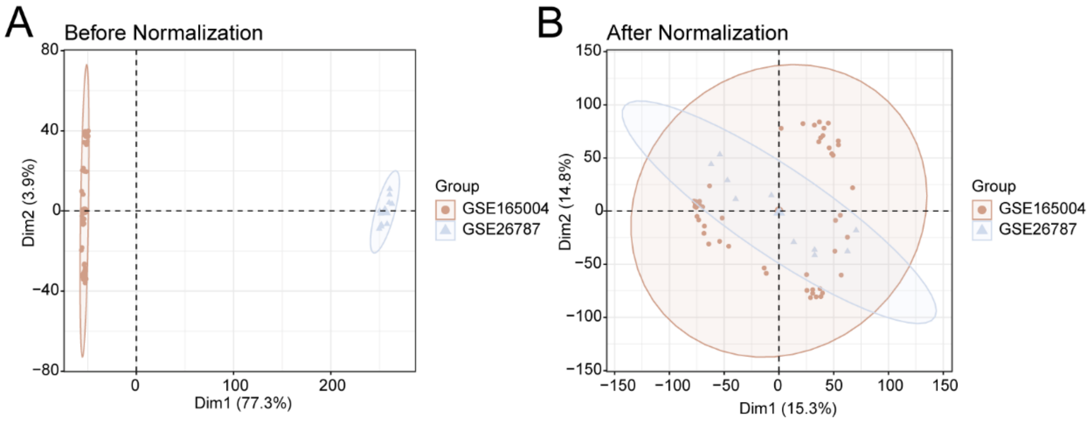

Fig. S1 Batch effects removal of GSE26787 and GSE165004

A. PCA diagram of the integrated dataset before batch removal. B. PCA diagram of integrated dataset after batch normalization. Brown-yellow for dataset GSE165004 and blue for dataset GSE26787. PCA: principal component analysis.

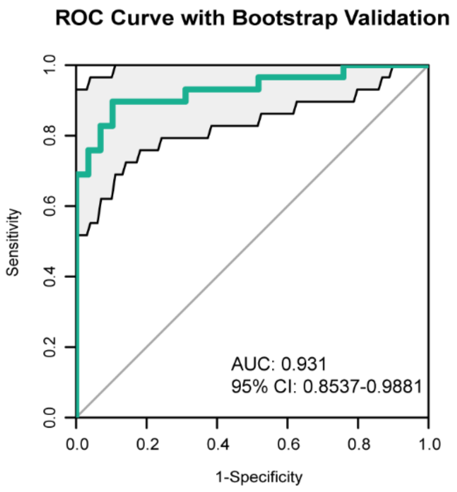

Fig. S2 Evaluation of the diagnostic model's performance and robustness

The 4-gene risk score demonstrated high accuracy in identifying RSA (AUC = 0.931). The robustness of the model was validated using 1,000 stratified bootstrap replicates, with the 95% CI (0.8537-0.9881) indicated by the shaded area. The diagonal dashed line represents a random classifier (AUC = 0.5).

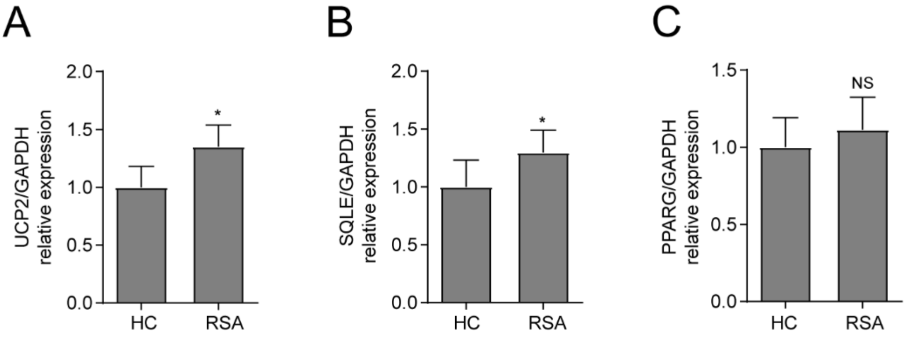

Fig. S3 qPCR analysis of *UCP2*, *SQLE* and *PPARG* expression in decidual tissues

A. Expression levels of *UCP2* in decidual tissues from women with RSA and healthy controls (HCs) were detected by qPCR. B. Expression levels of *SQLE* in decidual tissues from women with RSA and HCs were detected by qPCR. C. Expression levels of *PPARG* in decidual tissues from women with RSA and HCs were detected by qPCR. * $p < 0.05$. Data are expressed as the mean ± SD. NS: not significant.

Table S1 Quantitative PCR primer sequences used in this study

Target	Sequence
SREBF2	5′-ATGGGCAGCAGAGTTCCTTC-3′ (forward)
	5′-CGACAGTAGCAGGTCACAGG-3′ (reverse)
IGFBP1	5′-TTGGGACGCCATCAGTACCTA-3′ (forward)
	5′-TTGGCTAAACTCTCTACGACTCT-3′ (reverse)
PRL	5′-TGACCCTTCGAGACCTGTTTG-3′ (forward)
	5′-CTTGCTCCTTGTCTTCGGG-3′ (reverse)
GAPDH	5′-GGAGCGAGATCCCTCCAAAAT-3′ (forward)
	5′-GGCTGTTGTCATACTTCTCATGG-3′ (reverse)
UCP2	5′-GGGATTGACTGTCCACGCTC-3′ (forward)
	5′-ATACTATGTGTCCGAGCCGC-3′ (reverse)
SQLE	5′-GCCTGCCTTTCATTGGCTTC-3′ (forward)
	5′-TTCCTTTTCTGCGCCTCCTG-3′ (reverse)
PPARG	5′-AGTCAGCCTTTAACGAAATGACC-3′ (forward)
	5′-CACGGAGCTGATCCCAAAGT-3′ (reverse)

Table S2 Result of GO and KEGG enrichment analysis for model genes

ONTOLOGY	ID	Description	GeneRatio	BgRatio	*p*-value	*p*.adjust	*q*-value
BP	GO:0019915	lipid storage	3/4	86/18800	3.68478E-07	0.000140022	2.83146E-05
BP	GO:0010885	regulation of cholesterol storage	2/4	19/18800	5.7991E-06	0.000676466	0.000136792
BP	GO:0010878	cholesterol storage	2/4	21/18800	7.1207E-06	0.000676466	0.000136792
BP	GO:0071404	cellular response to low-density lipoprotein particle stimulus	2/4	21/18800	7.1207E-06	0.000676466	0.000136792
BP	GO:0055094	response to lipoprotein particle	2/4	33/18800	1.78882E-05	0.001159199	0.000234409
CC	GO:0012507	ER to Golgi transport vesicle membrane	1/4	62/19594	0.012597948	0.080738604	0.03642343
CC	GO:0030134	COPII-coated ER to Golgi transport vesicle	1/4	94/19594	0.019053348	0.080738604	0.03642343
CC	GO:0140534	endoplasmic reticulum protein-containing complex	1/4	125/19594	0.025276781	0.080738604	0.03642343
CC	GO:0030176	integral component of endoplasmic reticulum membrane	1/4	164/19594	0.033064144	0.080738604	0.03642343
CC	GO:0031227	intrinsic component of endoplasmic reticulum membrane	1/4	172/19594	0.034655767	0.080738604	0.03642343
MF	GO:0070888	E-box binding	2/4	49/18410	4.14978E-05	0.001369427	0.000174728
MF	GO:0008022	protein C-terminus binding	2/4	179/18410	0.000556874	0.009188415	0.001172366

MF	GO:0001227	DNA-binding transcription repressor activity, RNA polymerase II-specific	2/4	321/18410	0.001776791	0.010742761	0.001370687
MF	GO:0001217	DNA-binding transcription repressor activity	2/4	325/18410	0.001820885	0.010742761	0.001370687
MF	GO:0004955	prostaglandin receptor activity	1/4	10/18410	0.002171139	0.010742761	0.001370687
KEGG	hsa00100	Steroid biosynthesis	1/2	20/8164	0.004893857	0.049743084	0.014280311
KEGG	hsa05216	Thyroid cancer	1/2	37/8164	0.009044197	0.049743084	0.014280311
KEGG	hsa03320	PPAR signaling pathway	1/2	75/8164	0.018290066	0.057040959	0.016375395
KEGG	hsa04211	Longevity regulating pathway	1/2	89/8164	0.021685516	0.057040959	0.016375395
KEGG	hsa04152	AMPK signaling pathway	1/2	121/8164	0.029424454	0.057040959	0.016375395

GO: Gene Ontology, BP: biological process, CC: cellular component, MF: molecular function, KEGG: Kyoto Encyclopedia of Genes and Genomes.

Table S3 Results of GSEA for integrated dataset

ID	Description	setSize	Enrichment Score	NES	*p*-value	*p*.adjust	*q*-value
NABA_CORE_MATRISOME	NABA_CORE_MATRISOME	252	-0.429027385	-1.98073713	2.4679E-09	6.08832E-06	5.74891E-06
WP_COMPLEMENT_SYSTEM	WP_COMPLEMENT_SYSTEM	90	-0.576786188	-2.301594903	8.38758E-09	1.03461E-05	9.76933E-06
WP_GPCRS_CLASS_A_RHODOPSINLIKE	WP_GPCRS_CLASS_A_RHODOPSINLIKE	238	-0.4254679	-1.953474658	3.67448E-08	2.26623E-05	2.1399E-05
REACTOME_GPCR_LIGAND_BINDING	REACTOME_GPCR_LIGAND_BINDING	412	-0.360569422	-1.749616082	2.98249E-08	2.26623E-05	2.1399E-05
NABA_ECM_GLYCOPROTEINS	NABA_ECM_GLYCOPROTEINS	174	-0.448941961	-1.988321099	1.49122E-07	7.35769E-05	6.94753E-05
REACTOME_CLASS_A_1_RHODOPSIN_LIKE_RECEPTORS	REACTOME_CLASS_A_1_RHODOPSIN_LIKE_RECEPTORS	297	-0.378922928	-1.785032006	4.95707E-07	0.000203818	0.000192456
REACTOME_IMMUNOREGULATORY_INTERACTIONS_BETWE	REACTOME_IMMUNOREGULATORY_INTER	117	-0.494667197	-2.075265911	8.32454E-07	0.000256708	0.000242397

EN_A_LYMPHOID_AND_A_NON_LYMPHOID_CELL	ACTIONS_BETWEEN_A_LYMPHOID_AND_A_NON_LYMPHOID_CELL						
REACTOME_EXTRACELLULAR_MATRIX_ORGANIZATION	REACTOME_EXTRACELLULAR_MATRIX_ORGANIZATION	283	-0.375321547	-1.762874298	7.62497E-07	0.000256708	0.000242397
BIOCARTA_GHRELIN_PATHWAY	BIOCARTA_GHRELIN_PATHWAY	13	-0.856947442	-2.210400832	1.713E-06	0.000469551	0.000443375
PID_INTEGRIN2_PATHWAY	PID_INTEGRIN2_PATHWAY	27	-0.729825055	-2.284817311	3.18173E-06	0.000784933	0.000741176
WP_COMPLEMENT_SYSTEM_IN_NEURONAL_DEVELOPMENT_AND_PLASTICITY	WP_COMPLEMENT_SYSTEM_IN_NEURONAL_DEVELOPMENT_AND_PLASTICITY	98	-0.49137113	-2.006748194	4.98806E-06	0.001025462	0.000968296
KEGG_CYTOKINE_CYTOKINE_RECEPTOR_INTERACTION	KEGG_CYTOKINE_CYTOKINE_RECEPTOR_I	243	-0.380929708	-1.749802605	4.79942E-06	0.001025462	0.000968296

	NTERACTION						
WP_CHOLESTEROL_METABOLISM_WITH_BLOCH_AND_KANDUTSCHRUSSELL_PATHWAYS	WP_CHOLESTEROL_METABOLISM_WITH_BLOCH_AND_KANDUTSCHRUSSELL_PATHWAYS	43	0.634970933	2.159107943	2.16799E-05	0.003820305	0.003607337
KEGG_COMPLEMENT_AND_COAGULATION_CASCADES	KEGG_COMPLEMENT_AND_COAGULATION_CASCADES	67	-0.534411547	-2.020469943	2.06538E-05	0.003820305	0.003607337
WP_TYROBP_CAUSAL_NETWORK_IN_MICROGLIA	WP_TYROBP_CAUSAL_NETWORK_IN_MICROGLIA	58	-0.549607746	-2.017657366	3.07606E-05	0.004645222	0.004386268
WP_EXTRAFOLLICULAR_B_CELL_ACTIVATION_BY_SARSCOV2	WP_EXTRAFOLLICULAR_B_CELL_ACTIVATION_BY_SARSCOV2	67	-0.528536752	-1.998258882	2.98661E-05	0.004645222	0.004386268
WP_4249_HEDGEHOG_SIGNALING_PATHWAY	WP_4249_HEDGEHOG_SIGNALING_PATHW	40	-0.547249449	-1.878078671	0.000787286	0.048555851	0.045849038

	AY						
WP_MAPK_AND_NFKB_SIGNALING_PATHWAYS_INHIBITED_BY_YERSINIA_YOPJ	WP_MAPK_AND_NFKB_SIGNALING_PATHWAYS_INHIBITED_BY_YERSINIA_YOPJ	12	0.743552646	1.853021685	0.00225671	0.092538911	0.087380202
REACTOME_ERYTHROPOIETIN_ACTIVATES_PHOSPHOINOSITIDE_3_KINASE_PI3K	REACTOME_ERYTHROPOIETIN_ACTIVATES_PHOSPHOINOSITIDE_3_KINASE_PI3K	12	-0.728915529	-1.840666001	0.003101325	0.103391457	0.097627758
PID_HEDGEHOG_2PATHWAY	PID_HEDGEHOG_2PATHWAY	21	-0.585967261	-1.727907325	0.009134702	0.187176414	0.176742007

Table S4 Results of GSVA for integrated dataset

Pathway	logFC	AveExpr	*t*	*p*-value	adj.*p*.val	*B*
WP MAPK AND NFKB SIGNALING PATHWAYS INHIBITED BY YERSINIA YOPJ	0.739653	-0.00249	5.915758	9.67E-08	0.000289	7.525933
REACTOME NEUROTRANSMITTER CLEARANCE	-0.7123	-0.01088	-5.68407	2.49E-07	0.000372	6.660167
REACTOME DERMATAN SULFATE BIOSYNTHESIS	-0.66635	0.012755	-4.85257	6.64E-06	0.001988	3.665031
REACTOME AKT PHOSPHORYLATES TARGETS IN THE CYTOSOL	0.661004	0.013616	4.4691	2.79E-05	0.006417	2.359112
REACTOME CASPASE ACTIVATION VIA DEPENDENCE RECEPTORS IN THE ABSENCE OF LIGAND	0.655261	0.005492	4.92946	4.95E-06	0.001988	3.933298
WP RESOLVIN E1 AND RESOLVIN D1 SIGNALING PATHWAYS PROMOTING INFLAMMATION RESOLUTION	-0.64454	-0.00218	-4.85713	6.53E-06	0.001988	3.680884
WP INCLUSION BODY MYOSITIS	0.638578	0.001897	5.02546	3.42E-06	0.001988	4.270985
REACTOME ACETYLCHOLINE REGULATES INSULIN SECRETION	0.632695	0.009069	4.933845	4.86E-06	0.001988	3.94866
KEGG MEDICUS REFERENCE CA2 ENTRY LIGAND GATED CA2 CHANNEL	-0.62451	0.000283	-4.85184	6.66E-06	0.001988	3.66251
REACTOME BASE EXCISION REPAIR AP SITE FORMATION	0.602227	-0.02117	5.002213	3.74E-06	0.001988	4.188939

PID BETA CATENIN DEG PATHWAY	0.595675	0.022906	4.425926	3.27E-05	0.006515	2.215656
WP ERK PATHWAY IN HUNTINGTON 39 S DISEASE	0.594679	-0.02013	5.396732	7.93E-07	0.000789	5.603463
PID IL5 PATHWAY	-0.58807	0.005221	-3.76047	0.000338	0.024597	0.110117
KEGG MEDICUS REFERENCE MLK JNK SIGNALING PATHWAY	0.585962	-0.00337	4.262666	5.91E-05	0.010382	1.680262
REACTOME BRANCHED CHAIN AMINO ACID CATABOLISM	0.574993	-0.00036	3.943318	0.000182	0.016397	0.6676
REACTOME REGULATION OF RUNX1 EXPRESSION AND ACTIVITY	-0.57198	0.005234	-4.11951	9.83E-05	0.011547	1.220418
WP GLUCURONIDATION	-0.57032	0.00491	-4.21083	7.12E-05	0.010621	1.512699
KEGG ASCORBATE AND ALDARATE METABOLISM	-0.55974	0.009167	-4.22425	6.78E-05	0.010621	1.55595
REACTOME DISSOLUTION OF FIBRIN CLOT	-0.55862	-0.00868	-3.74739	0.000353	0.024651	0.070907
REACTOME FREE FATTY ACIDS REGULATE INSULIN SECRETION	0.555858	0.001729	3.885577	0.000221	0.017618	0.489718
